# Underestimated diversity in one of the world’s best studied mountain ranges: The polyploid complex of *Senecio carniolicus* (Asteraceae) contains four species in the European Alps

**DOI:** 10.11646/phytotaxa.213.1.1

**Published:** 2015-06-11

**Authors:** RUTH FLATSCHER, PEDRO ESCOBAR GARCÍA, KARL HÜLBER, MICHAELA SONNLEITNER, MANUELA WINKLER, JOHANNES SAUKEL, GERALD M. SCHNEEWEISS, PETER SCHÖNSWETTER

**Affiliations:** 1 Division of Systematics and Evolutionary Botany, Department of Botany and Biodiversity Research, University of Vienna, Rennweg 14, 1030 Vienna, Austria; michaela.sonnleitner@univie.ac.at, gerald.schneeweiss@univie.ac.at; 2 Institute of Botany, University of Innsbruck, Sternwartestrasse 15, A-6020 Innsbruck, Austria; peter.schoenswetter@uibk.ac.at; 3 Department of Botany, Natural History Museum, Burgring 7, A-1010 Vienna, Austria; pedro.escobar.garcia@univie.ac.at; 4 Division of Conservation Biology, Vegetation Ecology and Landscape Ecology, Department of Botany and Biodiversity Research, University of Vienna, Rennweg 14, Vienna, Austria; karl.huelber@univie.ac.at; 5 Vienna Institute for Nature Conservation & Analyses, Giessergasse 6/7, A-1090 Vienna, Austria; 6 GLORIA co-ordination, University of Natural Resources and Life Sciences Vienna, Center for Global Change and Sustainability & Austrian Academy of Sciences, Institute for Interdisciplinary Mountain Research, Silbergasse 30, A-1190 Vienna, Austria; manuela.winkler@boku.ac.at; 7 Department of Pharmacognosy, University of Vienna, Althanstrasse 14, A-1090 Vienna, Austria; johannes.saukel@univie.ac.at

**Keywords:** Asteraceae, European Alps, Polyploidy, *Senecio*, species nova

## Abstract

*Senecio carniolicus* (Asteraceae) is an intricate polyploid complex distributed in the European Alps (di-, tetra- and hexaploids) and Carpathians (hexaploids only). Molecular genetic, ecological, and crossing data allowed four evolutionary groups within *S. carniolicus* to be identified. Here, we establish that these four groups (two vicariant diploid lineages, tetraploids and hexaploids) are also morphologically differentiated. As a consequence, we draw taxonomic conclusions by characterizing four species, including the more narrowly circumscribed *S. carniolicus* (lectotypified here), the taxonomically elevated *S. insubricus* comb. nov. (lectotypified here), and the two newly described species *S. disjunctus* and *S. noricus*.

## Introduction

The European Alps are among the world’s best-explored mountain ranges with respect to their plant diversity, but additions to the inventory of Alpine plants still occur due to the discovery of species hitherto unknown from the Alps—e.g. *Saxifraga carpatica* Sternberg (1831: 32; published in Schneeweiss 1998) or *Carex glacialis* Mackenzie (1910: 244; published in Blanchemain *et al.* 2004)—or of so far overlooked species such as *Pinguicula poldinii* J. Steiger & Casper (published in Casper & Steiger 2001: 28), *Saxifraga styriaca* Köckinger (2003: 82) and *Alyssum neglectum* Magauer, Frajman & Schönswetter (published in Magauer *et al.* 2014: 500). Further additions to the Alpine flora resulted from disentangling the components of polyploid complexes composed of lower-ploid parental entities and their higher-ploid derivatives such as in the group of *Cardamine amara* Linnaeus (1753: 656), *Achillea pratensis* Saukel & R. Länger (1992: 160) or *Gymnadenia conopsea* (Linnaeus 1753: 942) R.Br. in W.T.Aiton (1813: 191) *s.l.* (Marhold 1992, Saukel & Länger 1992, Marhold *et al.* 2005).

*Senecio carniolicus* Willdenow (1803: 1993) has only recently been identified as an intricate polyploid complex (Suda *et al.* 2007, Sonnleitner *et al.* 2010). Within the long-recognised *Senecio* sect. *Jacobaea* (Miller 1754: 667) Gray (1821: 469), a monophyletic group of mostly western Eurasian species (Pelser *et al.* 2002, 2003), the species belongs to the informal *Incani*-clade containing mountain species distributed from the Spanish Sierra Nevada to the Carpathians. Delimitation and taxonomic status of *S. carniolicus* as well as its evolutionary relationships to close relatives have been discussed controversially. Although sometimes treated as subspecies of Western alpine *S. incanus* Linnaeus (1753: 869), (e.g., by Chater & Walters 1976, Aeschimann *et al.* 2004, Fischer *et al.* 2008), molecular data consistently support the distinctness of *S. carniolicus* (Pelser *et al.* 2003, Escobar García *et al.* 2012).

*Senecio carniolicus* is widely distributed in the Eastern European Alps and the Carpathians. The Alpine distribution area ranges from the Alpi Lepontine and the Prealpi Luganesi at the border between Switzerland and Italy to the easternmost central Alps of Austria (Suda *et al.* 2007, Sonnleitner *et al.* 2010). It occurs almost exclusively on siliceous bedrock (Ellenberg 1996) and thrives in a variety of alpine habitats, such as grasslands and dwarf shrub communities, stabilized scree slopes and rock crevices (Pignatti 1982), moraines and pioneer swards (Beger in Hegi 1928) as well as wind-exposed fellfields with strong freeze-thawing dynamics (Franz 1986). The altitudinal distribution ranges from timberline up to the nival zone (Reisigl & Pitschmann 1958).

In its previous circumscription *S. carniolicus* was considered a morphologically variable species, especially with respect to the leaf shape and indumentum. Plants from the western border of the species’ range with deeply divided and at least underneath densely hairy leaves have been described as *S. carniolicus* var. *insubricus* Chenevard (1906: 367). Later, this taxon was usually treated as *S. incanus* subsp. *insubricus* (Chenevard) Braun-Blanquet (1913: 300). It was commonly hypothesized that this entity, whose leaves possess some resemblance to those of *S. incanus*, might represent a hybrid between these two taxa (Chenevard 1906) with hypothetical intermediate ploidy (Ozenda *et al.* 1988). However, molecular data unambiguously show that this entity is most closely related to *S. carniolicus* with no traces of introgression by *S. incanus* (Escobar García *et al.* 2012). Additional infraspecific taxa of *S. carniolicus*-under *S. incanus* subsp. *carniolicus* (Willd.) Braun-Blanq. (1913: 300)—have been distinguished by Beger in Hegi (1928), who mentioned three, often coexisting *formae*: *S. incanus* f. *incanescens* A.Kern. ex Beger in Hegi (1928: 767) with greyish-tomentose indumentum, *S. incanus* f. *glabrescens* Hausm. ex Beger in Hegi (1928: 767) with glabrous to sparsely hairy leaves, and *S. incanus* f. *pinnatilobatus* Bornm. ex Beger in Hegi (1928: 767) with deeply incised leaves often with secondary lobes.

The presence of ploidy level variation in *S. carniolicus* (Schönswetter *et al.* 2007, Suda *et al.* 2007, Hülber *et al.* 2009) suggests that the morphological types might not merely be habitat-induced modifications of the same species, but instead correspond to different cytotypes. In the Alps, three main cytotypes (di-, tetra- and hexaploids) are found, while in the Carpathians only hexaploids occur (Suda *et al.* 2007). Cytotypes co-occur in major parts of the distribution area (Suda *et al.* 2007, Sonnleitner *et al.* 2010) sometimes within a few decimetres (Hülber *et al.* 2009, 2015). Nevertheless, intermediate ploidy levels were only found in ca. 1% of individuals in a comprehensive sample of about 5000 individuals (Sonnleitner *et al.* 2010), indicating strong crossing barriers under natural conditions. This is supported by molecular genetic differentiation among cytotypes (Hülber *et al.* 2015, M. Winkler *et al.* unpubl.) and, within the diploids, between two longitudinally vicariant groups (Escobar García *et al.* 2012); by poor seed sets and very low hybrid viability in crosses between (eastern) diploids and polyploids, though not between polyploids (Sonnleitner *et al.* 2013); and by microhabitat differentiation between the three main cytotypes (Sonnleitner *et al.* 2010, Hülber *et al.* 2015). Consequently, there is strong evidence that *S. carniolicus* in its present taxonomic circumscription contains four ecologically differentiated, genetically distinct, and partly reproductively isolated groups: western diploids, eastern diploids, tetraploids and hexaploids.

Here, we establish that these four groups are morphologically differentiated. As a consequence, we draw taxonomic conclusions by formally describing two new species and by redefining, at the species level, the circumscriptions of *S. carniolicus* subsp. *carniolicus* and *S. carniolicus* subsp. *insubricus*. In addition, we provide diagnostic characters and the geographic distribution of these four species as well as a determination key.

## Material and Methods

We sampled *S. carniolicus* in its current wide circumscription on 28 collecting sites evenly distributed over its distribution area (collecting sites 1, 2, 4, 10, 15, 18, 20, 21, 22, 23, 26, 40, 41, 46, 63, 64, 65, 66, 72, 77, 79, 80, 81, 87, 92, 96, 97, 100 in Sonnleitner *et al.* 2010), collecting one flowering shoot and one vegetative rosette per individual. As the collection was done without prior knowledge of the cytotype, sample sizes differ across the sites. Plant material was preserved in 75% alcoholic aqueous solution until preparation. Additionally, several specimens were taken from each population to be deposited in the herbarium WU. All individuals were ploidy-checked (Sonnleitner *et al.* 2010). From the alcohol-preserved material, one fully developed rosette leaf and one cauline leaf from the middle of the flowering stalk were pressed and dried. The number of ray and disk flowers of one fully anthetic capitulum was counted and flowers were mounted on sticky paper. Involucre width and length as well as the height of the flowering shoot and of the synflorescence were measured directly on the alcohol material. Values presented in the species descriptions correspond to the 10% and 90% quantiles, supplemented by extreme values scored on specimens listed under “Additional specimens examined”.

## Results and Discussion

The polyploid complex of *S. carniolicus* is an example of underestimated species diversity in the generally well-explored european Alps. It comprises a group of closely related taxa that are not only genetically and ecologically distinct, but exhibit also clear morphological differences. Our field experience showed that flowering individuals can be assigned to one of the four groups with high accuracy. Morphological separation of the two diploid taxa is mainly based on higher indumentum density and a lower number of flowering heads per synflorescence in western diploids, and coincides with complete geographical separation and the absence of recent gene flow (Escobar García *et al.* 2012). Polyploids differ from diploids in a taller growth and a longer corolla of ray flowers. In comparison to hexaploids, tetraploids are characterized by a stronger degree of the leaf dissection, i.e., deeply incised rosette leaves and presence of distinct secondary lobes in stem leaves.

The congruence of morphological differences with genetic divergence (Escobar García *et al.* 2012, Hülber *et al.* 2015, Winkler *et al.* unpubl.), ecological differentiation and the presence of crossing barriers (Schönswetter *et al.* 2007, Hülber *et al.* 2009, Sonnleitner *et al.* 2010, 2013) highlight the distinctness of these evolutionary lineages. On this basis we propose splitting *S. carniolicus* into four taxa, which together constitute the *S. carniolicus* agg. Separation at the species level seems most appropriate, because the four entities meet several requirements of different species concepts. Most importantly, reproductive isolation between diploids and polyploids is almost complete. No intermediate cytotypes were encountered in the broad area of co-occurrence of western diploids and hexaploids (Sonnleitner *et al.* 2010), and artificial crossings between eastern diploids and polyploids failed almost completely (Sonnleitner *et al.* 2013). Tetra- and hexaploids hybridize upon hand pollination (Sonnleitner *et al.* 2013), but intermediate cytotypes are nevertheless rare in nature (Sonnleitner *et al.* 2010). This is likely due to ecological differentiation particularly in areas of sympatry of tetraploids and hexaploids (Hülber *et al.* 2015). Specifically, tetraploids are most common on northern slopes, which are rarely occupied by hexaploids, and—in contrast to the other three entities—also extend to intermediate to slightly basic soils. Therefore, polyploids usually do not form mixed populations, but only narrow contact zones in areas of ecological overlap (Hülber *et al.* 2015). The four entities can therefore be regarded as functional biological species, which hybridize only occasionally. Occupation of different ecological niches or “adaptive zones” (Van Valen 1976), as demanded by the ecological species concept (Coyne & Orr 2004), further supports distinction at the species level.

Two taxa are newly described here, and *S. carniolicus* subsp. *insubricus* is raised to the species level with a new circumscription to comprise all diploids west of river Isel and south of river Drau. Application of the name *S. carniolicus* is restricted to the hexaploid cytotype. Due to the lack of morphological synapomorphies of the genus *Jacobaea* Miller (1754: 667), which was recently resurrected based solely on molecular evidence (chloroplast and ITS data; Pelser *et al.* 2006, 2007, Nordenstam & Greuter in Greuter & Raab-Straube 2006), we choose to make taxonomic changes and descriptions under *Senecio* Linnaeus (1753: 866), which is in accordance with recent taxonomic treatments (Pawłowski & Jasiewicz 1971, Aeschimann *et al.* 2004, Fischer *et al.* 2008, Calvo *et al.* 2015).

## Taxonomic treatment

### *Senecio carniolicus* aggregate

Perennial, herbaceous hemicryptophytes with short creeping rhizome, erect to ascending, lanulose stems, and alternate, spirally arranged leaves forming a basal rosette. Flowering stems with petiolate cauline leaves, which are reduced in size towards the apex. Capitula arranged in terminal cymose corymbs, heterogamous, radiate and yellow-flowered. Peduncules with one to three bracteoles in the upper part, lanulose. Involucre campanulate, with supplementary bracts; supplementary bracts few to several, narrowly linear to filiform; involucral bracts uniseriate, linear-lanceolate to narrowly oblong, attenuate, sparsely to densely tomentose, with dark reddish to blackish apex and somewhat longer bristles on the margin. Ray flowers female, fertile; tube cylindrical, lamina strap-shaped, apically three-toothed; disc flowers hermaphroditic; corolla narrowly tubular, gradually widening upwards, five-lobed, glabrous. Style branches apically obtuse with short sweeping-hairs. Pappus bristles simple, united in a basal ring, minutely barbellate with short acute teeth, off-white to yellowish or fawn-coloured, persistent. Achenes oblong, slightly flattened, longitudinally grooved, light brown, glabrous.

## Four species can be distinguished

**1. *Senecio carniolicus*** **Willdenow** (1803: 1993) ≡ *Jacobaea carniolica* (Willdenow) Schrank (1814: 316) ≡ *Senecio incanus* subsp. *carniolicus* (Willdenow) Braun-Blanquet (1913: 300) ≡ *Jacobaea incana* subsp. *carniolica* (Willdenow) B.Nord. & Greuter in Greuter & Raab-Straube (2006: 712).

**Type:**—sine loco, Herbarium Willdenow, B 15778/3! (lectotype, designated here); syntypes: sine loco, Herbarium Willdenow, B 15778/1; sine loco, Herbarium Willdenow, B 15778/2!; “Judenburger-Alpe, Im August 1811”, *Sieber*, Herbarium Willdenow, B 15778/5! The fifth syntype (“In alpibus Tolmiensibus”, Herbarium Willdenow, B 15778/4!) consists of plants with leaves densely hairy on both sides. This contradicts the diagnosis of Willdenow (“[…] Folia […] supra viridia subtus albido-pubescentia, juniora alba […]”). This specimen, which may have formed the basis for Willdenow’s epithet, is excluded from the original material because it cannot be the basis for the validating description; this specimen belongs to *S. insubricus*.

**FIGURE 1:**
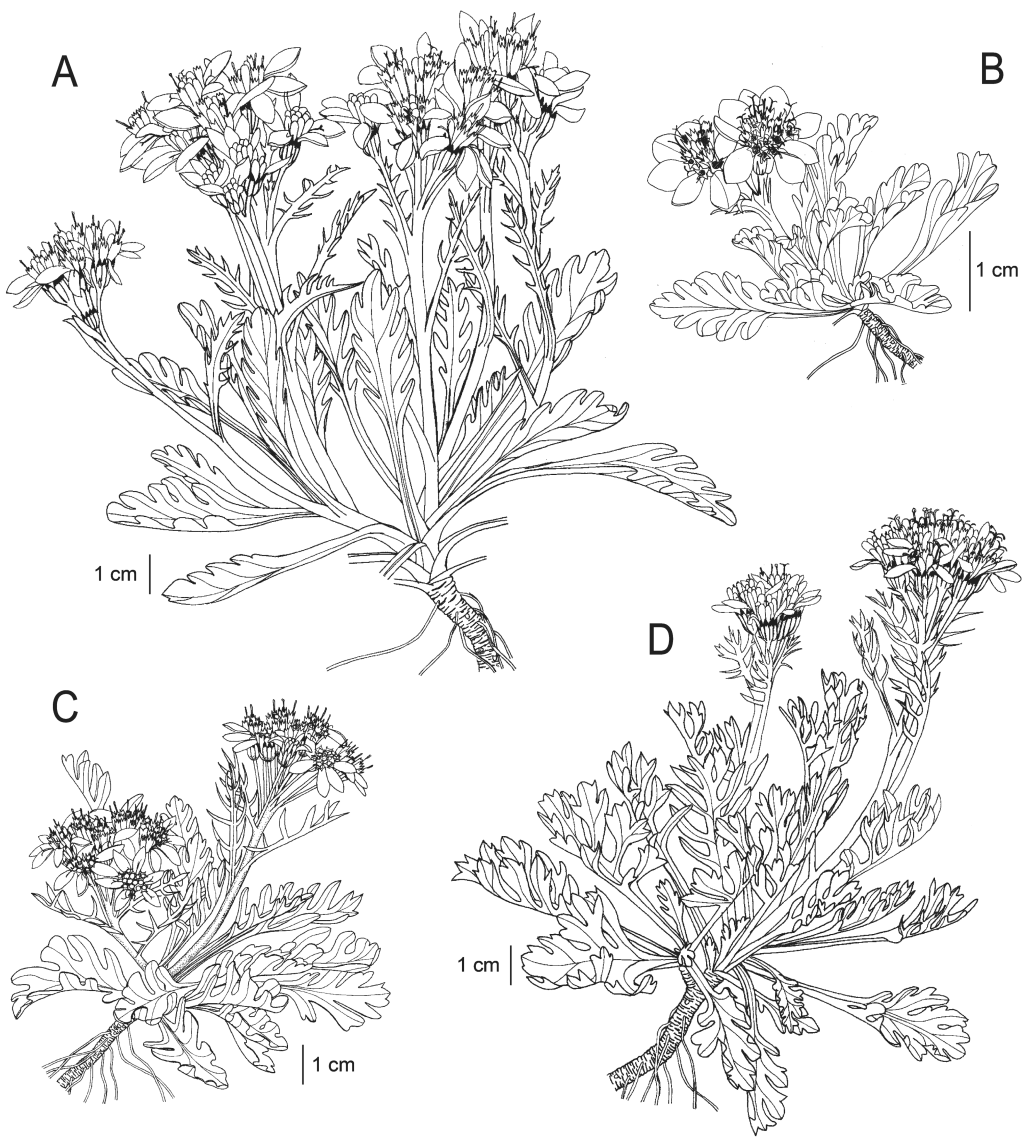
Iconography of *Senecio carniolicus* (A), *S. insubricus* (B), *S. noricus* (C) and *S. disjunctus* (D). Drawings: R. Flatscher.

**FIGURE 2:**
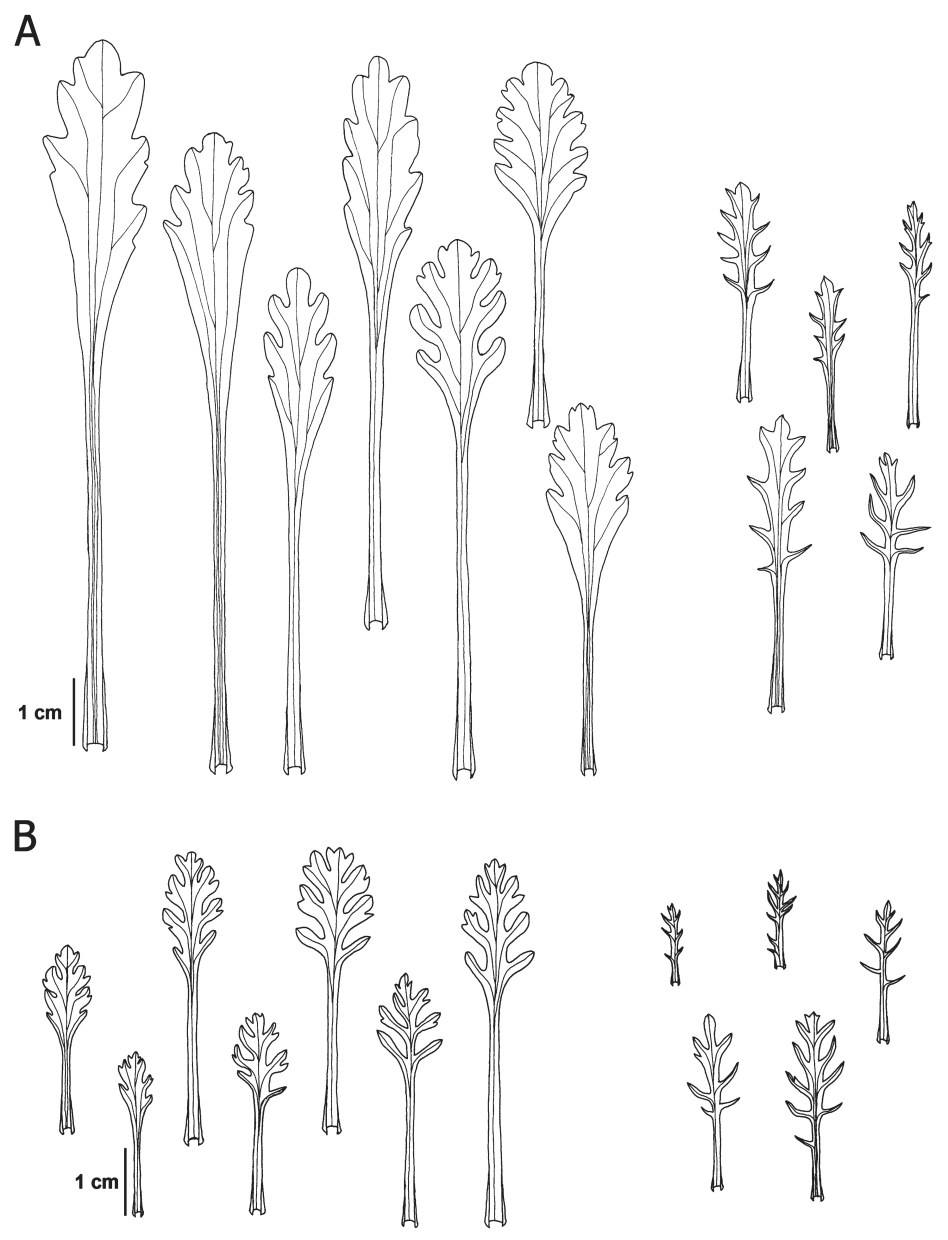
Representative shapes of rosette leaves (left) and cauline leaves (right) of *Senecio carniolicus* (A) and *S. noricus* (B). Drawings: R. Flatscher.

**Description:**—Plants (3)7–17(27) cm tall. Rosette leaves (4)5–10.5(13.5) cm long, petiolate, leaf blade (1.5)2–4.9(5.1) cm long, elongate-ovate to obovate in outline, with cuneiform base, shallowly lobed to dentate or almost entire, lobes usually only as long as wide, undivided, only rarely with smaller secondary lobes; young leaves densely tomentose and therefore greyish, upper leaf surface glabrescent with age, often subglabrous. Middle cauline leaves (1.3)2.7–4.8(6.2) cm long, sparsely tomentose to subglabrous. Capitula (5)6–14(23). Involucre (2.3)2.9–4(5.5) mm wide. Ray flowers (2)3–6(7), corolla (5.5)7–11.2(12.6) mm long. Disc flowers (5)7–14(16), corolla (4.6)5.6–7.4(8.2) mm long. Pollen grains (28)31–40(43) μm. Hexaploid (2*n* = 6*x* = 120). Figs 1A, 2A, 4A.

**Ecology:—**Alpine meadows, preferentially swards dominated by *Carex curvula* Allioni (1785b: 264), with high vegetation cover on siliceous substrate, ca. 1750–3150 m.

**Distribution:—**The species is distributed in the central Eastern Alps (Fig. 5A) from Rätische Alpen/Alpi Retiche and Bergeller Alpen/Monti della Val Bregaglia (Lombardia, Italy and Graubünden, Switzerland), Albula-Alpen and Silvretta (Graubünden, Switzerland as well as Vorarlberg and Tirol, Austria) eastwards to Gleinalpe (Steiermark, Austria). In addition, it occurs in high ranges of the Carpathians (Poland, Slovakia, Romania). It remains to be investigated if the morphologically slightly divergent plants from the Southern Carpathians (Romania) should be recognised as a separate entity.

**Etymology:—**The species is named after the historical Duchy of Carniola (Herzogtum Krain, Vojvodina Kranjska) within the Habsburg Empire, which comprised large parts of present-day Slovenia as well as southernmost Kärnten (Carinthia) and Steiermark (Styria). It should be noted that *S. carniolicus* does not occur in that area (see above under “Type”).

**2.** ***Senecio insubricus*** (Chenevard) R. Flatscher, Schneew. and Schönsw., **comb. et stat. nov.** ≡ *Senecio carniolicus* var. *insubricus* Chenevard (1906: 367) ≡ *Senecio incanus* subsp. *insubricus* (Chenevard) Braun-Blanquet (1913: 300) ≡ *Jacobaea carniolica* subsp. *insubrica* (Chenevard) Pelser (2006: 5) ≡ *Jacobaea incana* subsp. *insubrica* (Chenevard) B. Nord. & Greuter in Greuter & Raab-Straube (2006: 712).

**Type:**—[SWITZERLAND. Ticino/Tessin:] “Alpi di Pietra Rossa obenher Colla im Canton Tessin [Alpi di Pietra Rossa above Colla in the canton Tessin/Ticino], 20 July 1869”, *H. Siegfried*, ZT 37371! (lectotype, designated here); syntype: “Camoghe”, *O. Heer*, ZT 37373!

**FIGURE 3:**
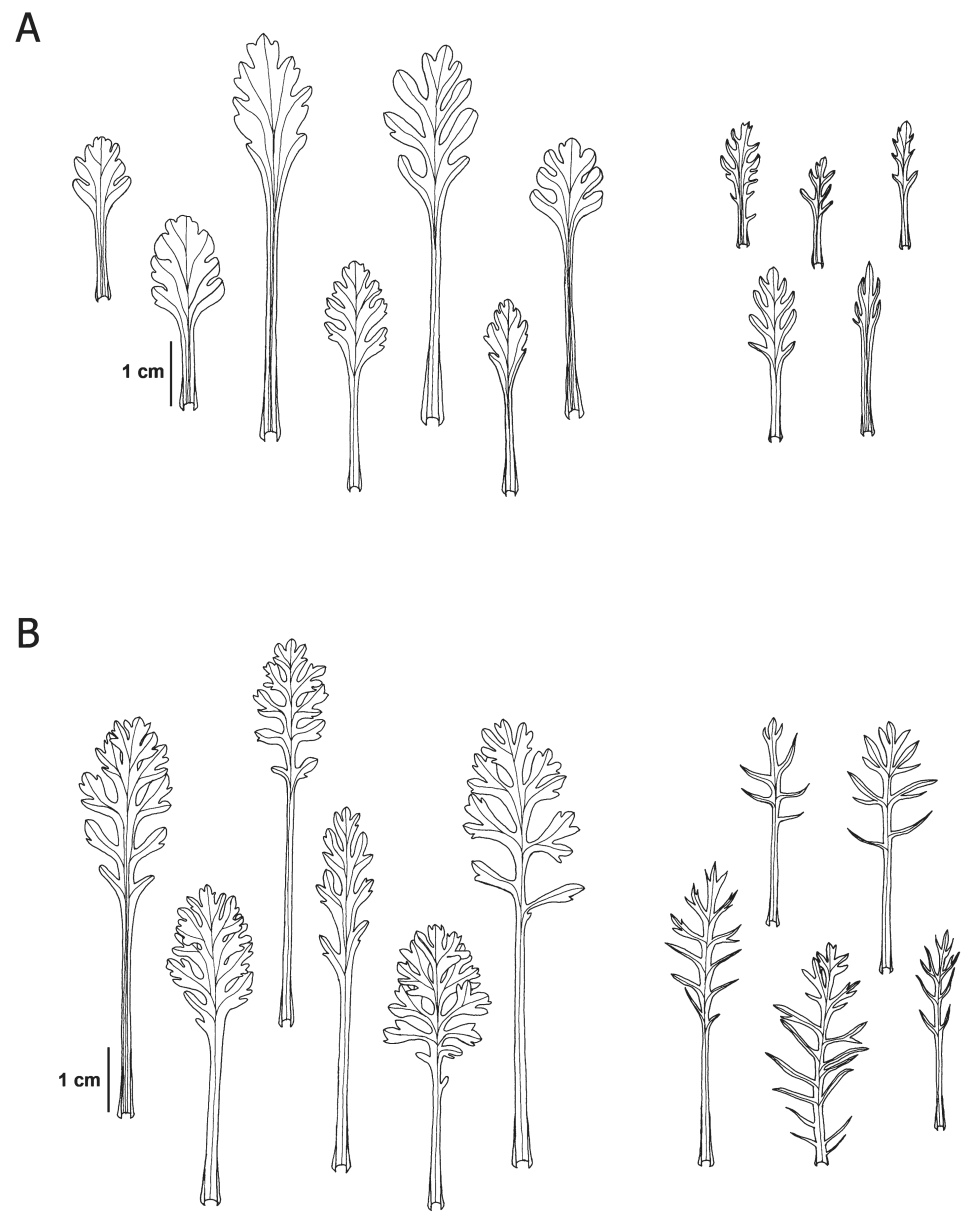
Representative shapes of rosette leaves (left) and cauline leaves (right) of *Senecio insubricus* (A) and *S. disjunctus* (B). Drawings: R. Flatscher.

**FIGURE 4:**
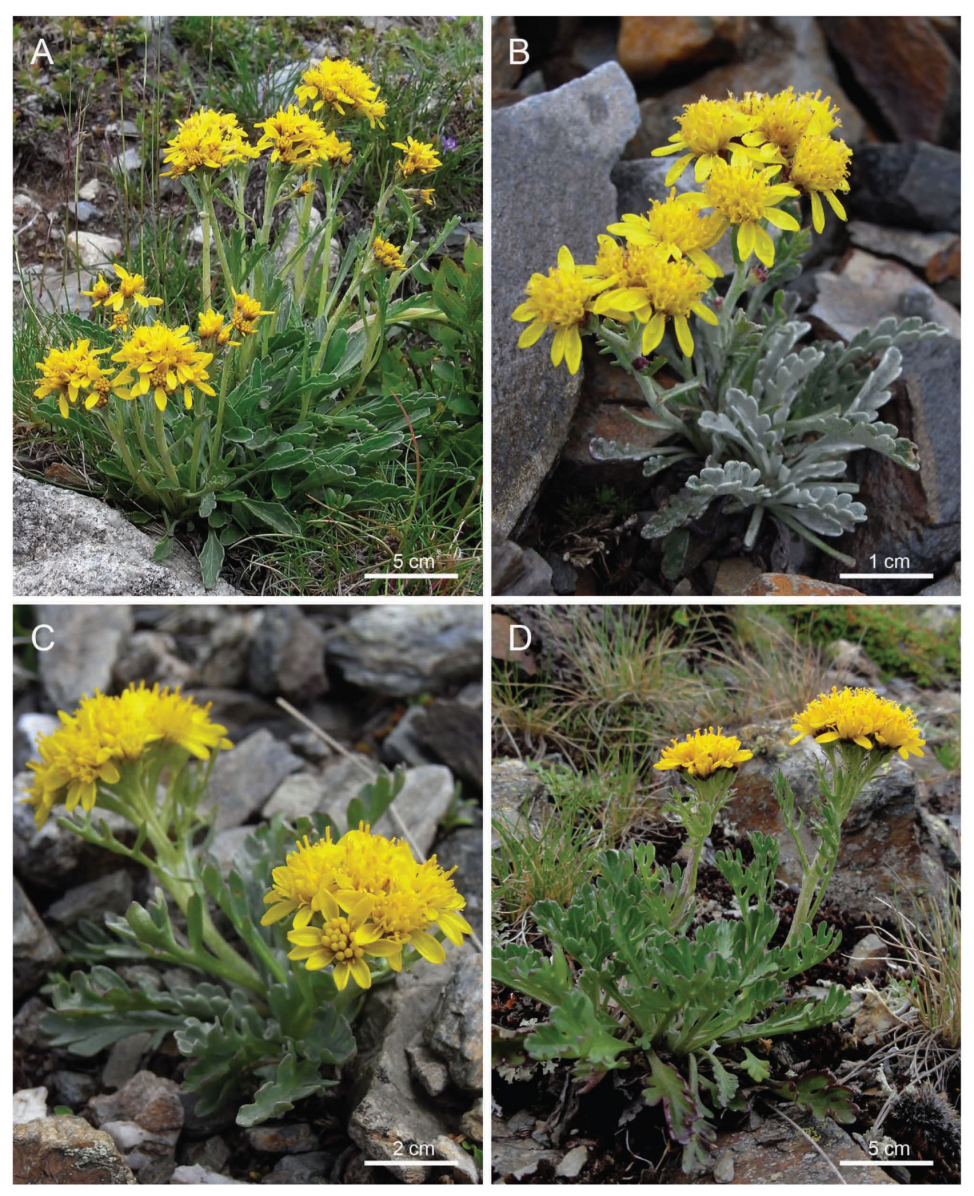
Representative individuals of *Senecio carniolicus* (A, Almerhorn, population 58 from Sonnleitner *et al.* 2010), *S. insubricus* (B; Plose, population 46), *S. noricus* (C; Bretthöhe, population 80), and *S. disjunctus* (D; Bretthöhe, population 80). Note the characteristic differences in indumentum density and leaf dissection as well as in the number of capitula per synflorescence. Photographs: M. Sonnleitner.

**Description:**—Plants (3)4–9(15) cm tall. Rosette leaves (2.2)2.6–6.5(8.7) cm long, petiolate, leaf blade (1)1.5–2.5(3.5) cm long, ovate in outline, usually with truncate to rounded base, lobed to deeply incised. Middle cauline leaves (1.1)1.6–3.4(5.7) cm long. All leaves densely and persistently tomentose on both sides, whitish, rarely single leaves glabrescent. Capitula 2–5(6). Involucre (2.2)2.9–4.4(4.6) mm wide. Ray flowers (3)4–8, corolla (5)6–8.5(9.6) mm long; disc flowers (10)12–22(25), corolla (4.3)4.6–6.1(6.4) mm long. Pollen grains (26)27–32(37) μm. Diploid (2*n* = 2*x* = 40). Figs 1B, 3A, 4B.

**Ecology:—**Rock crevices and other habitats in the alpine to subnival zone with shallow soil layer on siliceous bedrock, very rarely also on carbonates (most prominently, the disjunct population in the Karawanken/Karavanke thrives on dolomite); ca. 1850–3150 m.

**FIGURE 5:**
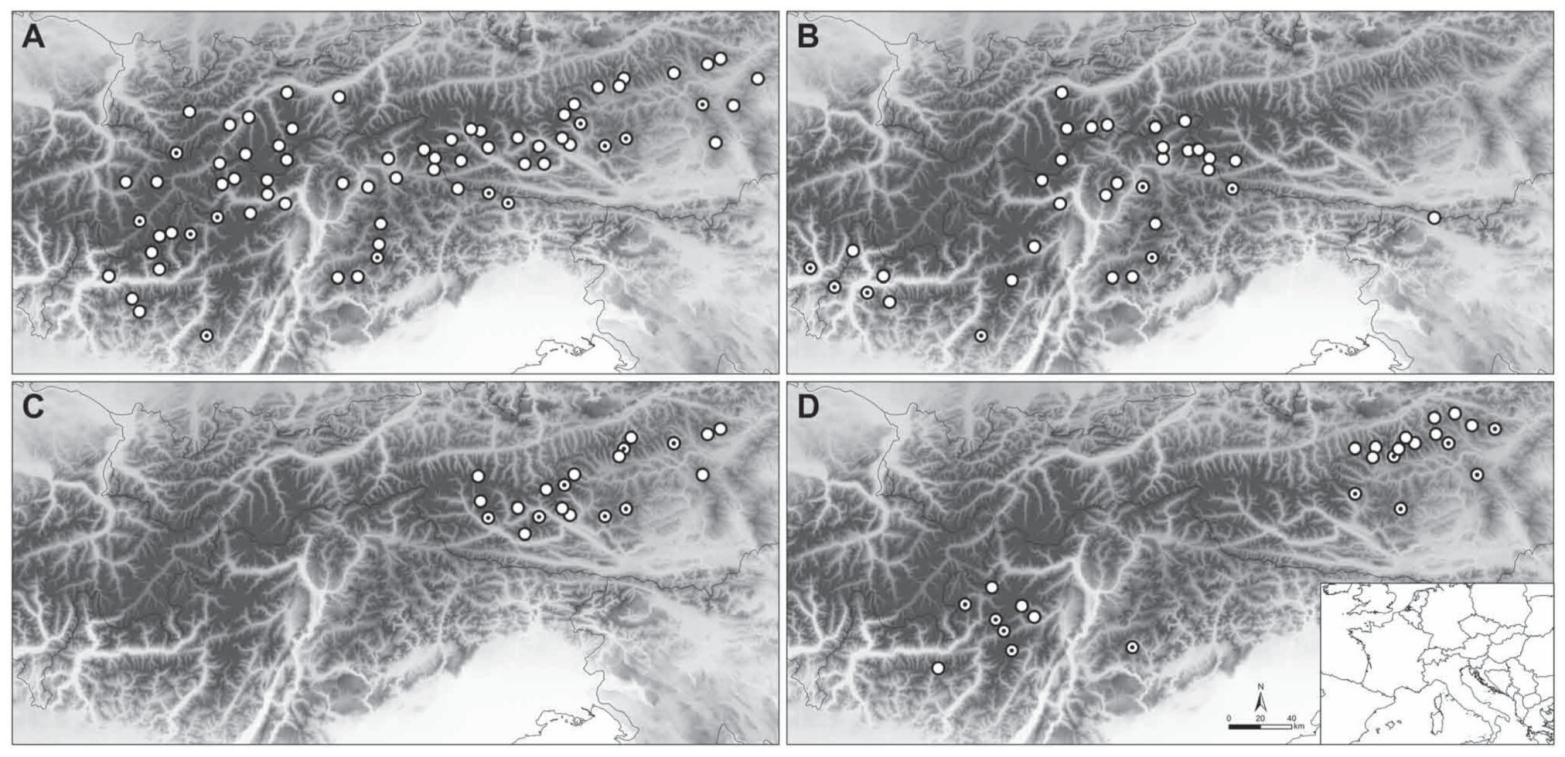
Distribution of the four species of the *Senecio carniolicus* agg. in the Eastern Alps based on Sonnleitner *et al.* (2010); *S. carniolicus* (A), *S. insubricus* (B), *S. noricus* (C) and *S. disjunctus* (D). Morphometrically evaluated populations are marked with a black dot.

**Distribution:—**The species occurs in the western part of the central Eastern Alps (Fig. 5B) from the Alpi Lepontine (Ticino, southern Switzerland/Italy) to the Isel Valley (Osttirol, Austria); disjunct occurrences are in the southeastern Alps in the Karnische Alpen/Alpi Carniche (Austria, Italy) and the Karawanken/Karavanke (Kärnten, Austria/Gorenjska, Slovenia).

**Etymology:—**The species takes its epithet from a historical region in Northern Italy between Lago di Como and Lago di Garda. The name of the region is connected to the ancient tribe of the “insubres” mentioned by several ancient Roman authors. Chenevard first used this epithet in his description of a novel variety of *S. carniolicus.* He considered this taxon a possible hybrid of *S. carniolicus* with the vicariant *S. incanus,* with a narrowly endemic distribution confined to the Alpi Lepontine and Alpi Orobie.

**3.** ***Senecio noricus*** R. Flatscher, Schneew. and Schönsw., **sp. nov.**

**Type:—**AUSTRIA. Salzburg, Ankogelgruppe, Hohe Tauern, Großer Hafner, 47°4‘9‘‘N, 13°23‘45‘‘E, ca. 2890 m, 13 August 2008, *P. Escobar García* (holotype WU 0080559!; isotypes WU, W, IB, GZU, Z, and BOZ).

**Description:—**Plants (2.7)3.8–8.1(9.3) cm tall. Rosette leaves (1.8)2.5–6(8) cm, petiolate, leaf blade (1)1.5–2.5(3) cm long, ovate in outline, usually with truncate to rounded base, lobed to deeply parted, sparsely hairy, greyish-green. Middle cauline leaves (1.1)1.4–3.3(3.6) cm, petiolate. Capitula (4)6–13(16). Involucre (2)2.2–3.2(3.7) mm wide. Ray flowers 3–7(8), corolla (4.3)4.6–6.7(7.5) mm long. Disc flowers (8)10–14(16), corolla (3.9)4.4–5.9(7.2) mm long. Pollen grains (23)25–31(35) μm. Diploid (2*n* = 2*x* = 40). Figs 1C, 2B, 4C.

**Diagnosis:**—*Senecio noricus* differs from the other members of the *S. carniolicus* agg. in the following characters: leaves not persistently densely hairy on both sides, therefore not whitish (vs. *S. insubricus*); plants usually 4–8 cm tall, pollen grains (23)25–31(35) μm in diameter, growing in open, exposed habitats (vs. *S. disjunctus* and *S. carniolicus*).

**Ecology:—**The species thrives in open, exposed habitats, such as alpine fellfields, on siliceous bedrock, and occurs from ca. 2000 to 3000 m.

**Distribution:—**The species is endemic to Austria and occurs in the eastern part of the central Eastern Alps (Fig. 5C) from the Isel Valley (Osttirol) to the easternmost Niedere Tauern (Seckauer Alpen, Steiermark, Austria).

**Etymology:—**The species takes its name from the province Noricum in the Ancient Roman Empire, which comprised the current Austrian federal states Ober- and Niederösterreich, Kärnten and Steiermark as well as parts of Tirol.

4. ***Senecio disjunctus*** R. Flatscher, Schneew. and Schönsw., **sp. nov.**

**Type:**—AUSTRIA, Steiermark, Rottenmanner Tauern, Großer Bösenstein, 47°26‘24‘‘N, 14°24‘48‘‘ E, ca. 2110 m, 27 July 2008, *P. Escobar García* (holotype WU 0080561!, isotypes WU, W, IB, GZU, Z and BOZ).

**Description:**—Plants (4.6)5.3–16.1(19.2) cm tall. Rosette leaves (2.5)3.5–8.5(11) cm long, petiolate, leaf blade (1.6)2–4(5) cm long, ovate in outline, usually with truncate base, deeply lobed to pinnatisect, lobes usually more than two times longer than wide, usually with one or more small distinct secondary lobes, glabrous in individuals occupying the eastern partial area, sparsely hairy to subglabrous in individuals from the western partial area. Middle cauline leaves (1.6)2.1–4.5(7.4) cm long. Capitula (4)6–15(21). Involucre (2.5)2.8–4(4.9) mm wide. Ray flowers (3)4–7(10), corolla (3.9)6.1–8.8(9.4) mm long. Disc flowers (8)10–18(21), corolla (2.5)4.9–6.9(7.2) mm long. Pollen grains (26)28–36(37) μm. Tetraploid (2*n* = 4*x* = 80). Figs 1D, 3B, 4D.

**Diagnosis:—***Senecio disjunctus* differs from other members of the *S. carniolicus* agg. in the following characters: leaves not persistently densely hairy on both sides, therefore not whitish (vs. *S. insubricus*); plants usually 5–16 cm tall, pollen grains (26)28–36(37) μm in diameter, usually growing in habitats with dense vegetation cover (vs. *S. noricus*); rosette leaves deeply incised, lateral lobes usually longer than wide, always divided (vs. *S. carniolicus*).

**Ecology:—**Alpine meadows and dwarf shrub communities, with a tendency towards north-exposed slopes, sometimes also on stony, more shallow soils than *S. carniolicus*; usually on siliceous bedrock, but more frequently found on intermediate to slightly basic substrates than the other species; ca. 1870–3080 m.

**Distribution:—**The species occurs in two disjunct distribution areas (Fig. 5D); the western partial range spans from the Alpi Bergamasche to the Ortler/Ortles and Adamello massifs (Südtirol/Trentino/Brescia, Italy; Graubünden, Switzerland) and the eastern partial range extends from the easternmost Hohe Tauern eastwards (Salzburg/Kärnten/Steiermark/Austria). It remains to be investigated based on a broader sampling whether the morphological differences between individuals from the two partial distribution areas are constant enough to allow for the taxonomic recognition at the subspecific level.

**Etymology:—**The epithet refers to the distribution pattern of the species, whose range is split into two disjunct partial areas.

## Determination key to the *S. carniolicus* aggregate and its closest relatives

This key includes all Alpine species of the *Incani*-clade sensu Pelser *et al.* (2003) with the exception of the morphologically very divergent *S. abrotanifolius* Linnaeus (1753: 869). Thus, in addition to the *S. carniolicus* aggregate this key also contains *S. incanus*, which was formerly often considered conspecific with *S. carniolicus* (e.g., Chater & Walters 1976, Aeschimann *et al.* 2004, Fischer *et al.* 2008), and the morphologically clearly distinct and thus taxonomically uncontroversial Western Alpine species *S. persoonii* De Notaris (1844: 229) and *S. halleri* Dandy (1970: 625) (≡ *S. uniflorus* (Allioni 1773: 70) Allioni (1785a: 200), non Retzius (1783: 42).
1Well-developed plants with one single large (20–25 mm in diameter) capitulum per flowering stem***S. halleri***Note: Hybridizes with *S. incanus* in areas where both species co-occur (Wilczek 1900); these hybrids are morphologically intermediate.-Well-developed plants with two or more smaller (10–15 mm in diameter) capitula per flowering stem, arranged in a terminal synflorescence22Ray flowers absent***S. persoonii***-Ray flowers present33All leaves white-greyish tomentose, rosette leaves deeply incised with narrow lateral segments; achenes pubescent on the upper part***S. incanus***-All leaves green and subglabrous to white-greyish tomentose, rosette leaves shallowly lobed or with almost entire margin to deeply incised, but never in the combination described above; achenes glabrous**4 (*S. carniolicus agg.*)**4Leaves densely persistently hairy, whitish; 2–5(6) capitula per flowering stem***S. insubricus***
-Leaves glabrous to sparsely hairy, green or greyish, or young leaves densely hairy and older leaves glabrescent; (4)6–15(23) capitula per flowering stem**5**
5Flowering stem (2.7)3.8–8.1(9.3) cm long; corolla of ray flowers (4.3)4.6–6.7(7.5) mm long; pollen grains (23)25–31(35) μm in diameter; grows in open, exposed habitats, fellfields, rock crevices; eastern part of the central Eastern Alps in Austria***S. noricus***-Flowering stem (3.4)5.3–17.3(27) cm long; corolla of ray flowers (3.9)6.1–11.2(12.6) mm long; pollen grains (26)28–40(43) μm in diameter; grows in alpine meadows, dwarf shrub communities and other habitats with dense vegetation cover; throughout most of the distribution of the *S. carniolicus* aggregate**6**6Rosette leaves deeply (more than half the distance to the midrib) incised, lateral lobes longer than wide, always divided (usually with one or two secondary lobes), adult leaves of eastern (Austrian) populations glabrous, those of western (Italian and Swiss) populations pubescent***S. disjunctus***-Rosette leaves shallowly lobed (less than half the distance to the midrib) to dentate or rarely almost entire, lateral lobes at most as long as wide, only occasionally with secondary lobes, hairy to slightly tomentose at least when young, older leaves often glabrescent***S. carniolicus***


## Appendix 1 Specimina visa. This list includes specimens kept at IB, W, and WU. Text added by us is in squared brackets

### Senecio carniolicus

#### Austria

[Salzburg], felsige Matten des Speiereck im Lungau; *F. Vierhapper*, August 1897, WU.—[Salzburg], Preber im Lungau; *F. Vierhapper*, 4 August 1873, WU.—[Salzburg], Speiereck im Lungau; *F. Vierhapper*, 9 August 1873, WU.—[Steiermark], Judenburg: am Zirbitzkogel bei ca. 1700 m; *E. Khek*, 30 July 1898, W.—[Steiermark], Seethaler Alpen ad Judenburg. 1700–2390 m, solo schistoso; *B. Przybylsky*, 15 August 1889, W.—1 km O-Seilbahn-Bergstation, Patscherkofel; A. *Ch. Mrkvicka*, 6 July 2000, W.—Alp. Seethal; –, s.d.; Herb. *Reichenbach W*.—Alpe Kühlbrein bei Turrach, Steiermark; *Jos. Ahrenberger*, July 1867, W.—Alpen Niedere Tauern, Judenburger Alpen, Zinggen; *Fenzl*, s.d., W.—Alpentriften am Abhang des Gsteinsjoch gegen das Pflumtal (Ferwallgruppe) Nord-Tirol; *H. Zerny*, 30 July 1919, W.—Alpenwiesen oberhalb Sagritz in Kärnthen; *D. Pacher*, September, W.—Alpes Styriae Superioris am Hohen Zinken; *Welwitsch*, s.d., W.—am Hohen Zinken bei Seckau; *H.W. Reichardt*, 12 August 1861, W.—am Kamm der Saualpe bei Kärnthen; *Woloszczak*, 3 August 1866, W.—am Kreuzeck bei Greifenburg in Oberkärnten. Urschiefer, 2700 m; *E. Preissmann*, August 1863, W.—am Patscherkofel bei Innsbruck; *M. Přichoda*, August 1880, W.—Arlberg, Passhöhe, 1802m; *K. Ronniger*, 26 June 1937, W.—Arlberggebiet: nächst der Ulmerhütte, 1900 m; *Rudolf Berger*, 18 August 1921, IB-15100.—auf dem Hundsfeldkopf nächst dem Radstätter Tauern, 6200–7600‘; *O. Simony*, 4 August 1863, W.—auf dem Rothkogel nächst Turrach im Steiermark; *Dr. H.W. Reichhardt*, 14 August 1865, W.—auf dem Rücken der Gleinalpen; *J. B*., 27 July 1868, WU.—auf der Gleinalpe bei Übelbach in Steiermark–Glein; *E. Preissmann*, 9 July 1891, W.—auf Fels und im Gerölle der Judenburger Alpen; *Gebhard, Welwitsch*, August, W.—auf sämtlichen Malteiner Alpen; *B. Kohlmayr*, s.d., WU.—auf Schiefern am Wege vom Turracher See auf das Ochsenbrett, ca. 6000 Fuß, Kärnthen; *Jos. Ahrenberger,* 3 August 1870, WU.—aufwärts von der Trogalpe gegen die Spitze des Speiereck bei Mauterndorf, Schiefer, 2200 m; *Louis Keller*, 20 August 1895, W.—Bachlenke, Defregger Alpen, Tirol; *J. Schneider*, 26 July 1929, W.—bei Mallnitz: auf Alpentriften am Aufstieg vom Seebachthal Hannoverhaus und in dessen Umgebung, 2300 m; *Hirth*, 12 August 1924, W.—bei Mallnitz: auf Alpentriften am Weg zur Lonzahöhe; *Hirth*, 21 July 1924, W.—Berger Thörl, üppige Alpenwiesen gegen Kals, 2200–2300 m; *Ronniger*, 29 July 1890, W.—Bockhartscharte Felsen; *O. Wittmer*, 6 August 1930, W.—Brandstätter Graben; *W. Gutermann*, 29 July 2006, WU.—Bundesland Salzburg, Radstädter Tauern, Obertauern, nahe der Passhöhe, alpine Silikatrasen [um 1740 m]; *Peter Buchner*, August [19]75, W.—Carinthia; *Hauser*, s.d., W.—Carinthia, auf der Pasterze am Großglockner; –, 6 August; Herb. Reichenbach fil. W.—Carinthia, Hohes Winterthal; *D. Pacher*, July-August, W.—Carinthia, Reichenauer Garten; *D. Pacher,* August, W.—Eisenhut (Häufig) 1904, Hochreichert (Gipfel 2417m) nied. Tauern, 1905; *Dr. Hel. Zellner*, s.d., W.—Falkert Moschelitzen im Nockgebiet; *M. A. Fischer*, 25 July 1966, WU.—felsige Abhänge im oberen Moosthale bei St. Anton am Arlberge, Schiefer, 2000 m; *E. Preissmann*, 16 July 1900, W.—Fimberjoch; ex Herb. *Peyritsch*, August 1986, IB-15069.—Finstertal bei Umhausen; ex Herb. *Peyritsch*, 20 July 1883, IB-15073.—Glimmerschieferfelsen auf der Arlberg-, 1800 m; *E. Preissmann*, 12 July 1901, W.—Glungezer; –, 23 July 1911, IB-15092.—Gmeineck bei Gmünd (Kärnten), nicht Häufig, ca. 2300 m, Kalk und Urgestein; *Louis Keller*, 4 September 1903, W.—Grau-Kogel, Gastein in Salzburg; *Pappitz*, 15 July1841, W.—Gregerlenock, Schoberriegel, Turracher Höhe; *Melanie Pull*, July 1966, W.—Gregerlnock bei Turrach; *B. Fest*, July 1905, WU.—Gregerlnock bei Turrach, Urgebirge, 1960 m; *Schule Frojach*, 28 July 1907, W.—Groß Vermunt Alp (Montafon); *H. Kemp*, 3 August 1871, W.—Großer Reichart; *Wolosrzczak*, 8 August 1876, W.—Heiligenbluter Tauern, Carinthia; –, s.d.; Herbar Putterlick W.—Hirnkopf, ca. 1800 m bei Flattnitz; *Melanie Pull*, 1 August 1973, W.—Hochschwung; *Julius Nevole*, August 1907, WU.—Hohe Lorenzispitz–Obernberg; *Ebner*, s.d., WU.—Hohe Tauern. Hochalmspitzgruppe. Säuleck. Knapp unter dem Gipfel; *Schönbeck*, 6 August 1948, WU.—Hohe Tauern. Schleinitz b. Lienz, Neualpensee, Almboden; *Schönbeck*, 3 August 1964, WU.—Hohenwart, Niedere Tauern, Steiermark; *Julius Nevole*, August 1906, WU.—Hornthalerjoch zwischen Sellrain und Stubai; *Kerner*, s.d., WU.—in alpibus prope H[eiligen]blut; *Hoppe*, s.d., W.—in alpibus Styriae superioribus, Judenburgeralpen; *Welwitsch*, s.d., W.—in Alpibus Styriae, Carniolica; Herb. Porthenschlag, s.d., W.—in alpibus summis Carinthiacis pr. H[eiligen]blut; *Dr. Hoppe*, s.d., W.—in der Nähe des Redtenbacher-Gletschers über d. Pitzthaler Jöchl zur Braunschweiger-Hütte, nicht hfg., Urgestein; *Louis Keller*, 24 August 1902, W.—in graminosis lapidosis alpium prope forum Seckau, ca. 1800 in solo; *Dr. Pernhoffer*, August 1894, WU.—in Sch[reihuber?]alpe prope H[eiligen]blut; –, s.d., W.—Innsbruck, bei 7000’; *Glanz*[?], s.d., W.—Judenburger Alpe; *Sieber*, August 1811, W.—Judenburgeralpen und Kärnthner Alp.; *Gebh. Moser*[?], s.d., W.—Kaernthen: Sau-Alpe; *J. Wiesbauer*, August 1861, W.—Karnische Alpen: Obertilliacher Tal zw. Talschluss (1527 m) und Porzescharte (ca. 2500 m); *Neumayer*, July 1937, W.—Kärnten, Bergwiesen auf der Turracher Höhe; *Johann Vetter*, 29 July 1932, W.—Kärnten, Gurktaler Alpen, Feldhütte–Naßbodenhütte–Rosennock, 1758–2440 m; *P. Schönswetter & A. Tribsch* (3747), 20 August 1999, WU.—Kärnten, Gurktaler Alpen, Innerkrems: Stubennock, Gipfelbereich, Silikatrasen, 2080 m; *M. Strudl*, 11 September 1978, W.—Kärnten, Hohe Tauern: Goldberggruppe, Melenböden–über NW-Hang weglos auf den (Gr.) Sadnig–Sadnigscharte–Mulleten Sadnig–Hirtenkof–Striedenkopf–Scharte gegen Makernispitz, 2200–2745 m; *Andreas Tribsch* (5597), 9 July 2000, WU.—Kärnten, Hohe Tauern: Goldberggruppe, Sadniggruppe, Grossfragant, Melenböden, 0,7 km ESE der Sadnigscharte, 2280 m; *Gerald M. Schneeweiß* (11147), 6 July 2006, WU.—Kärnten, Hohe Tauern: Hafnergruppe, Gmeinalm–Torscharte–Poisnig–Wandspitze, 2000-2623 m; *P. Schönswetter & A. Tribsch* (4633), 13 July 2000, WU.—Kärnten, Leitertal am Bergerthörl, Häufig, Urgestein ca. 2600 m; *Louis Keller*, 5 August 1899, W.—Kärnten, Sadnig; *Roland Albert*, July 1982, W.—Kärnten, Saualpe; *Roland Albert*, 27 September 1973, W.—Kärnten, Saualpe, Offnerhütte–Ladinger Graben–Ladinger Spitz–Gertrusk, 1700–2079 m; *P. Schönswetter & A. Tribsch* (4564), 3 July 2000, WU.—Kärnten, Saualpe, Umgebung der Weißberger Hütte (6–8 km östlich oberhalb Wieting), ca. 1600–1800 m; *H. Malicky*, September 1975, WU.—Kärnten, Wolayer-See-Gebiet; *Heinricher*, August 1924, IB-15085.—Kärnten-Villach: auf grusigem Boden am Gipfel der Gerlitzenalpe; *Dr. Korb*, 7 August 1929, W.—Kärnthen. am Uebergang von der Stangalpe nach dem Winkel Reichenau; *Dr. Korb*, 4 August 1907, W.—kiesige Wiese am Gregerle Nock bei Turrach in Steiermark; *Seiler*, July 1914, W.—Kleinalpe, 6200’; *Fürstenwerther*, 30 July 1854, W.—Kühtai in Tirol 1966 m; *J. Witasek*, 11 August 1909, WU.—Lungau (Salzburg), Aineck; *F. Vierhapper*, ca. 1900, WU.—Lungau (Salzburg), Bundschuh; *F. Vierhapper*, s.d., WU.—Lungau (Salzburg), Kaareck; *F. Vierhapper*, s.d., WU.—Lungau (Salzburg), Lasaberg; *F. Vierhapper,* ca. 1900, WU.—Lungau (Salzburg), Matten des Pleisnitzkogel im Murwinkel; *F. Vierhapper*, August 1898, WU.—Lungau (Salzburg), Mühlbacher Nock; *F. Vierhapper*, ca. 1900, WU.—Lungau (Salzburg), Preber; *F. Vierhapper*, ca. 1900, WU.—Lungau (Salzburg), Reiseck; *F. Vierhapper*, ca. 1900, WU.—Lungau (Salzburg), Speyereck; *F*. *Vierhapper*, s.d., WU.—Mallnitzer Tauern, Kärnten; *J. Schneider*, 15 July 1929, W.—Montafon, Verwallgruppe, Sennigrat, Bergstation des Sesselliftes; *Gölles*, 24 July 1988, WU.—Monte “Fassal”, 8000’, Vorarlberg; *Rehsteiner*, *Helvet*., August 1849, W.—Nassfelder Tauern; *Ruprecht*, s.d., WU.—N-Kärnten: Hochalmgruppe, Großelend oberhalb der Osnabrückerhütte; *K. Ronniger*, 30 July 1907, W.—Nockberge, Gruft (3 km SE der Turracher Höhe); *Gölles*, 17 August 1988, WU.—nördlicher Abhang des Finstertaler Sch. bei Umhausen im Ötzthal; *Handel-Mazzetti*, am 25 July 1895, WU.—Nordtirol, am Venet, oberhalb Landeck, vom Krahberg zur Glanderspitze, ca. 2200–2300: Almwiesen, Felsheiden, Felsen, Zwergstrauchheiden, Silikat; *F. Krendl*, 23 August 1985, W.—Nordtirol, Diasalpe, Höhenweg zum Niederelbehaus, Ferwall; *K. Fitz*, 12 July 1949, W.—Nordtirol, Lechtaler Alpen: SW Lech, von der Monzabon Alpe nach Zürs, ca. 1700–1980 m, Almwiesen, Quellfluren, Felsen, Unterlage: Kalk; *F. Krendl*, 11 August 1982, W.—Nordtirol, Lechtaler Alpen: vom Rüfikopf über die Bockbach-Scharte zur Stuttgarter Hütte, ca. 2300–2360 m, Almwiesen, Schutthalden, Felsen, Schneeböden, Kalk; *F.Krendl*, 12 August 1982, W.—Nordtirol, Ötztaler Alpen: Pitztal: zwischen Mittelberg und der Braunschweiger Hütte, 2510 m; *A. Gilli*, 18–30 July 1977, W.—Nord-Tirol, Patscherkofel östl. der Schutzhütte, 2100 m, Gleisglimmerschiefer; *Rudolf Berger*, 20 August 1919, IB-15103.—Nord-Tirol, Silvrettagruppe, ober der Jamtalhütte, 2500 m.; *Paul v. Stark*, August [19]23, IB-15102.—oberes Gailtal, Berge um Obertilliach; *H*. *Neumayer*, 1 August 1908, WU.—Obergurgl, Gurgler Heide, 2200 m. Alpine Rasen; *I.* & *S. Bortenschlager*, July 1973, IB-39598.—Osttirol, Defereggen-Gebirge: von der Michelbachalm zur Weißen Wand; *F. Krendl*, 2 August 1969, W.—Osttirol, Hohe Tauern, Kalser Tauern, Schutthang; *G. Malicky*, 2 August 1966, W.—Osttirol, Karnische Alpen: Rollertal bei Obertilliach, ca. 1600 m, auf höheren Silikat-Alluvionen des Baches; *A. Polatschek*, 2–15 September 1979, W.—Osttirol, Lesachtal, Karnische Alpen SSW von Obertilliach, Obertilliacher Tal, Tilliacher Joch, 2150 m; *E. Vitek*, 9 August 1994, W.—Ötztaler Alpen: am Aufstieg Braunschweigerhütte-Pitztaler Jöchl, Silikat, 8 August 1910; *Karl Ronniger*, 8 August 1910, W.—Ötztaler Alpen: Am Aufstieg Vent-Breslauer Hütte, Silikat; *Karl Ronniger*, 18 August 1910, W.—Ötzthal, Fend; *Karl Grimus v. Grimburg*, July 1884, W.—Padaster im Gschnitztal, 2200 m; *Sarnthein*, s.d., WU.—Pasterze; –, s.d.; Herb. Reichenbach fil. W.—Patscher Kofl; *J. Murr*, 1889, IB-15074.—Patscher Kovel; *J. Amprosi*, s.d., WU.—Patscherkofel bei Innsbruck; *Kerner*, July 1875, WU.—Patscherkogel bei Innsbruck; *F. Hofmann*, August 1880, W.—Peischelkopf ca. 2300 m [Verwallgruppe]; *G. Richen*, 6 July 1897, W.—Pillerhöhe, Montavon, ca. 2000 m; *J. Rhomberg*, 21 July 1885, W.—Pinzgau, am Kapruner Thörl; *Karl Aust*, Sommer 1883, W.—Preber, Tamsweg (2400 m); *J. Schneider*, 22 August 1902, W.—Rätikon am Schlappin[er Joch] [S v. Garganellen]; *C. Boetzkes*, 9 September 1869, W.—Raurisergoldberg; *F.Vierhapper*, 14 August 1872, WU.—Salzburg, Hohe Tauern, am Schiefergerölle auf dem …cherboden des Hohen Goldberges; *Dr. Korb*, 18 August 1905, W.—Salzburg, Hohe Tauern: Goldberggruppe, N-Grat des Kalkbretterkopf (3.5 km S der Türchlwand), 2200–2412 m; *P. Schönswetter & A. Tribsch* (3711), 12 September 1999, WU.—Salzburg, Hohe Tauern: Umgebung der Neuen Rudolfshütte, Eisboden; *F. Kummert*,17 August 1971, W.—Salzburg: Unter feuchten Felsen in der Großarl und Kleinarl, Schiefer; *Heinrich Freiherr von Handel-Mazzetti*, 24–26 July 1900, WU.—Saualpe; *Schiffer*, s.d., W.—Saualpe im Granitgebirge; –, s.d.; Herb. Reichenbach fil. W.—Saualpe Triften 2000 m; *Jabornegg*, s.d., WU.—Sch…. spitz ober der Längenthaler Alphütten gegen Praxmar im Sellrain; *Kerner*, 1872, WU.—Schleinitzalpe Lienz, Tirol; *Pappitz*, 28 August 1843, W.—Seckauer Zinken, n Steyermark, Gneiss; *Wolosrzczak*, 4 August 1876, W.—Seckauer Zinken, Steiermark; *J. Breidler*, 12 August 1865, WU.—Seckauer Zinken, Steiermark; *G. Kraskovits*, s.d., WU.—Seckaueralpen; *Haß..ann*, s.d., W.—Speikkogel der Gleinalpe; *Wettstein*, s.d., WU.—Speikkogel der Gleinalpe; –, 1882, WU.—Steiermarck, Abhang westlich vom Turracher See; *F. Buxbaum*, 23 August 1922, W.—Steiermark Seckauer Alpen Zinken; *G. Sennholz*, 19 July 1887, W.—Steiermark Zinken bei Seckau; *Leod. Derganc*, August 1894, WU.—Steiermark, Gleinalpe, Gasthof Gleinalm–Speikkogel, 1800–1988 m; *Andreas Tribsch* (5229), 13 September 2000, WU.—Steiermark, Lavanttaler Alpen, Seetaler Alpen, Zirbitzkogel, ca. 500-600 m SW oberhalb vom gr. Winterleitensee, am Ostfuß des Kreiskogels (SW Judenburg), 1900–1960 m, felsige Stellen zwischen Zwergstrauchheiden über Silikat; *E. Hörandl, F. & F. Hadaček, B. Wallnöfer*, 15 August 1991, W.—Steiermark, Niedere Tauern, Seckauer Zincken, Aufstieg vom Pabstriegel über Kote 1869 zum Gipfel, 1950–2330; *F. Kummert*, July 1971, W.—Steiermark, Packalpe, Hirschegger Sattel–Speikkogel–Hofalmkogel–Ameringkogel, 1700–2187 m; *Andreas Tribsch* (5220), 12 September 2000, WU.—Steiermark, Seckauer Alpen, Lamprechthöhe–Schweigerhöhe, 2000–2214 m; *Andreas Tribsch* (5218), 11 September 2000, WU.—Steiermark, Seetaler Alpen, Lindersee–Gipfel des Zirbitzkogel, 2051–2396 m; *P. Schönswetter & A. Tribsch* (3735), 12 August 1999, WU.—Steiermark, Wölzer Tauern, Schießeck, wenig E von P. 2275, 2240 m; *Gerald M. Schneeweiß & Peter Schönswetter* (12466), 21 August 2008, WU.—Steiermark. auf dem Hochzinken bei Seckau; *Thomas Pichler*, comm. *Dr. v. Eichenfeld*, s.d., W.—Steiermark. Auf dem Hohen Zinken bei Seckau; *Th. Pichler*, August 1875, WU.—Steiermark: auf steinigem Boden auf der Stangalpe bei Turrach; *Johann Vetter*, 22 July 1924, W.—Steinige Matten des Gamsspitz im Weißbriach; *F. Vierhapper*, August 1899, WU.—steirisch-kärntnerisches Grenzgebiet, Alpenmatten auf dem Rinsennock bei Turrach; *Johann Vetter*, 7 August 1924, W.—steirisch-kärtnerisches Grenzgebiet. im niederen Grase auf Alpenmatten am Königsstuhl bei Turrach; *Johann Vetter*, 2 August 1924, W.—Stiria superior, Alpes Zinken prope Seckau, 7500’; *J.C. Eques Pittoni a Dannenfeld*, 19 September 1853, W.—Stiria, Hoher Zinken Altitudo 5000’. Im Gerölle des Glimmerschiefers; –, et. comm. *Eq. de Pittoni*, W.—Stiria, Hoher Zinken, Altitudo 5000’, Im Gerölle des Glimmerschiefers; *Eq. de Pittoni*, 15 August, W.—Stiria, Hoher-Zinken, 5000’, im Gerölle des Glimmerschiefers; *Eq. de Pittoni*, 15 August, W.—Stiria, Stubalpe Speikkogel; *J.C. Eques Pittoni a Dannenfeld*, 5 August 1842, W.—Styria. Hohen-Zinken; –, August; Herb. Fenzl ex Herb. Equitis de Pittoni W.—Styria. Hohen-Zinken; *Eq. de Pittoni*, August, W.—Tirol Daumbühel am Aufstiege zur Kreithspitze (Heute Greitspitze) über Ranalt im Stubaithale; *Kerner*, 1869[?], WU.—Tirol Daumbühel am Aufstiege zur Kreithspitze (Heute Greitspitze) über Ranalt im Stubaithale; –, 1869[?]; Herb. *Kerner* WU.—Tirol Daumbühel am Aufstiege zur Kreithspitze (Heute Greitspitze) über Ranalt im Stubaithale; –, 1869[?]; Herb. *Kerner* WU.—Tirol Ötzthal; *Kerner*, 1874, WU.—Tirol Ötzthaler Alpen zwischen Vent und der Ramolalpe; Ginzberger Zederbauer, s.d., WU.—Tirol Trins; *F. Wettstein*, s.d., WU.—Tirol, Lienzer Dolomiten (Gailtaler Alpen p. p.), Connyalm–Kutteschupfen–Golzentipp, 2070–2317 m; *P. Schönswetter & A. Tribsch* (4602), 6 July 2000, WU.—Tirol, Ötztaler Alpen, Gaisbergtal (SSE Obergurgl): bis knapp vor den Gaisbergferner und östliche Talflanken (v. a. gegen P. 3049 südlich des Festkogels), 2200–3049 m; *P. Schönswetter & A. Tribsch* (3690), 30 August 1999, WU.—Tirol, Ötztaler Alpen, Wegabzweigung auf ca. 2560 von der Kaunertaler Gletscherstraße–P. 3044 N über dem Weißseejoch, 2560–3044 m; *P. Schönswetter & M. Wiedermann* (5208), 16 September 2000, WU.—Tirol, Samnaungruppe, Fisser Joch–Zwölferkopf–Oberer Sattelkopf–Hinterer Sattelkopf, 2430–2670 m; *P. Schönswetter & A. Tribsch* (4935), 13 August 2000, WU.—Tirol, Schleinitz bei Lienz, auf den höchsten Jochen in Felsen und Spalten; *Th. Pichler*, 5 September, W.—Tirol, Tuxer Alpen, Tulfeinalm–Tulfeinjoch–Glungezer (über die E-Flanke), 2035–2677 m; *P. Schönswetter & M. Wiedermann* (5187), 14 September 2000, WU.—Tirol, Vikarspitze [Viggarspitze] bei Innsbruck; *F. Höpflinger*, 9 August 1947, IB-15067.—Tirol, Villgratner Berge, Kuschletalm–Pfanntörl–Toblacher Pfannhorn, 2000–2663 m; *P. Schönswetter & A. Tribsch* (4594), 5 July 2000, WU.—Tirol, Villgratner Berge, Lipper Alm–Kalksteiner Jöchl, 1900–2326 m; *P. Schönswetter & A. Tribsch* (4596), 5 July 2000, WU.—Tirol, Zillertaler Alpen, S unterhalb des Saurüssel, 2600–2700 m; *Peter Schönswetter & Katharina Bardy* (12129), 1 September 2007, WU.—Tirol. Großglockner. Auf Alpentriften auf dem Bergertörl; *Dr. Ernst Korb*, 25 July 1907, W.—Tirol. im kurzen Grase und im Gebüsch zwischen zwischen dem Schönbichele und dem bösen Weibele bei Lienz; *Johann Vetter*, 11 August 1922, W.—Tirol/Osttirol, Hohe Tauern: Granatspitzgruppe, Bergstation des Goldriedliftes–Kals-Matreier-Törl–Kalser Höhe–Weißer Knopf; Kals-Matreier-Törl–gegen den Gorner (bis 2500 m s. m.), 2146–2577 m; *P. Schönswetter & A. Tribsch* (4881), 7 August 2000, WU.—Tirol/Osttirol, Rieserfernergruppe/Villgratener Berge, Obersee NE des Staller Sattel, 2020–2060 m; *P. Schönswetter & A. Tribsch* (4894), 8 August 2000, WU.—Tirol/Vorarlberg, Lechtaler Alpen, Gipfel der Valluga–Trittkopf–Trittscharte–Ulmer Hütte–Arlensattel–E-Flanke des Galzig, 2057–2809 m; *P. Schönswetter & M. Wiedermann* (5191), 14 September 2000, WU.—Tirol/Vorarlberg, Silvretta, Bieler Höhe–Hohes Rad, 2100–2934 m; *P. Schönswetter & M. Wiedermann* (5196), 15 September 2000, WU.—Tirol: Auf Bergwiesen nächst dem Kals-Matreier Törl; *Johann Vetter*, 6 August 1909, W.—Tirol: Fels nächst der Vernagthütte bei Rofen im Oetztal; *Johann Vetter,* 14 August 1919, W.—Tirol: von Mittelberg zur Braunschweigerhütte im Pitztal; *Louis Keller*, 10 August 1906, W.—Tirolia centralis. In monte Patscherkofel ad Innsbruck 7000’; *Kerner*, s.d., W.—Turracher Höhe, Steiermark; *General J. Schneider*, 28 July 1921; W.—Turracherhöhe; *J. Schneider*, 10 July 1935, W.—Versailspitze [in der Verwall-Gruppe]; *G. Richen*, 27 August 1897, W.—Verwallgruppe; zw. St. Christoph am Arlberg und Kaltenberger Hütte, 2080 m; *Alexander Gilli*, August 1969, W.—Vikarspitze [Viggarspitze]; *Friedrich Stolz*, 31 August 1894, IB-15091.—vom Hohen Zinken; *Dr. Fenzl*, s.d., W.—vom Hohen Zinken im Steiermark; *Pittoni*, 1843, W.—von der Pasterze; –, s.d.; Herb. Reichenbach fil. W.—von Heiligenblut via Sonnblick nach Gastein; –, 20 August 1894, WU.—Vorarlberg, Allgäuer Alpen: Hoher Ifen: Pellingers Köpfle; *E. Dörr*, 27 July 1986, W.—Vorarlberg, Arlberghöhe; *J .Murr*, 1895, W.—Vorarlberg, Verwall-Gruppe: Sattelkopf S Wald/Arlberg, ca. 1850 m, auf Silikatschutt in der Zwergstrauchheide; *A. Polatschek*, 6 August 1978, W.—Vorarlberg, Verwall-Gruppe: Wormser Höhenweg, zwischen Wormser Hütte und Valschavieler Maderer Grat, ca. 2100–2300 m; Weiden, Felsen, Felsheiden, Silikat; *F. Krendl*, 2 September 1986, W.—Zentralalpen: Ötztaler Alpen Obergurgl Rotmoostal; *Alois F. Geyrhofer*, s.d., WU.—Zillertaler Alpen, Tuxerjoch, 2000 m, Urgestein; *Rudolf Berger*, 21 July 1920, IB-15090.—Zirbitzkogel, Seetaler Alpen, St; J. Schneider, 11 August 1925, W.—zw. Hüttenkogel–Graukogel–Palfner Hochalm, 2230–2000 m, Silikat; *Mück*, 13 August 1968, W.

#### Italy

(Unterhornhaus) Rittnerhorn, Tirol, 2044 m, grasige Triften (Porphyr); –, 30 July 1917; ex Herb. Diettrich-Kalkhoff IB-15082.—2250 m hoch auf Dolomit am Langkofeljoch bei St. Ulrich in Gröden; *W. Hecht*, 10 August 1910, W.—Alpe Padon im Bellunesischen an der Gränze von Fassa; –, s.d.; Herb. Reichenbach fil. W.—Alpen des Pusterthales; *Bainer[? or Lainer?],* 18[??], WU.—Alpen des Pusterthales; *Stainer* (herb. Kerner), s.d., IB-15068.—Alpen um Bozen; v. *Grabmayr*, s.d., WU.—Alpi di Bolzano; *Viehweider*, s.d., WU.—am Stilfser Joch von 9200–7000 Fuß abwärts, blühen zwischen 8000 und 9000 Fuß; *O. Simony*, 29 August 1855, W.—auf dem …zuge zwischen Panereggio und S. Pellegrino bei 6000‘; *Fuchs*, s.d., W.—auf den Porphyrbergen zwischen Paneveggio und S. Pelegrino in Tirol, in einer Höhe von 6500–7000‘; –, s.d., W.—auf der Spitze des Saß de Capell bei Canazei im Faßa-Thale, Porphyr (?), 6000‘; *Fenzl*, 6 September 1867, W.—Bagolino, Lombardei, 2000 m; *J.Dörfler*, 1897, W.—Belluno, Dolomiten, Passo Pordoi–Rifugio Sass Beccei–Col de Cuch–Sattel vor Cresta Larice, 2250–2563 m; *P. Schönswetter & A. Tribsch* (3638), 26 August 1999, WU.—Brescia, Gardaseeberge, Monte Colombine, 2200–2214 m; *P. Schönswetter & A. Tribsch* (3662), 28 August 1999, WU.—Cima d‘Asta-Gebiet; Nordgipfel des Cengello; *Handel-Mazzetti*, 26 August 1906, WU.—Eggentaler Alm/Südtirol, Augit-Porphyrit-Fels westlich des Satteljochs, 2270 m. „Windkante“: niederer Rasen mit Kobresia myosuroides; *Klaus Vorhauser*, 5 August 1996, IB-15066.—Fassa; *Bernard*, August 1864, WU.—Fassa Thal; *Pappitz*, 5 August 1841, W.—Felsabhänge an der Stilfserjochstrasse bei Bormio, 1800 m; *J. Preissmann*, 3 August 1900, W.—Fravort, Levico; *J. Schneider*, 24 July 1909, W.—Friuli-Venezia-Giulia: Udine, Karnische Alpen, Monte Crostis (SW Plöckenpaß): S-Kamm und Nordabstürze, 1900–2250 m; *P. Schönswetter & A. Tribsch* (4574), 4 July 2000, WU.—Helm; –, 26 July 1902, W.—Helm; *M.F. Müllner*, 4 September 1879, W.—im Madritschtal von 7500 bis gegen 8500‘; *O. Simony*, 1855, W.—in alpibus editioribus Tyrol. austr. Schlern. Ritten; *Hausmann*, 1831, W.—Laugenspitze bei Meran; *Leybold*, s.d., W.—Lombardia: Sondrio, Bernina-Alpen, (Pra Sücc)–Tre Cornini–Bivacco Bottani Cornaggio–P. 2636–gegen Monte Spluga, 2000–2700 m; *Andreas Tribsch* (4957), 14 August 2000, WU.—Lombardia: Sondrio, Bernina-Alpen, Lago di Campo Moro–Bochetta delle Forbici–nördliche Umgebung der Bochetta delle Forbici, 2050–2750 m; *Peter Schönswetter* (4948), 14 August 2000, WU.—Lombardia: Sondrio, Livigno-Alpen, Forcola di Livigno–La Stretta–Punta la Stretta, 2315–3104 m; *P. Schönswetter* (5055), 21 August 2000, WU.—Lombardia: Sondrio, Livigno-Alpen, Monte Rocca, 1,8 km NNE Passo di Foscagno, 2700–2800 m; *Andreas Tribsch* (5092), 24 August 2000, WU.—Lombardia: Sondrio, Livigno-Alpen, Umgebung der Forcola di Livigno–M. Vago, 2300–3059 m; *Andreas Tribsch* (5043), 21 August 2000, WU.—Lombardia: Sondrio, Livigno-Alpen, Val Verva–Sasso di Castro–gegen Monte Verva (ca. 12 km WSW Sondrio), 2250–2750 m; *Andreas Tribsch* (5100), 25 August 2000, WU.—Lombardia: Sondrio/Bergamo, Bergamasker Alpen, Valle Lunga SW Tartano: Laghi di Porcile–Passo di Porcile–Cima Cadelle, 2000–2483 m; *Peter Schönswetter* (4996), 18 August 2000, WU.—Longobardia superioris, Prov. di Sondrio, Bormio, in rupestribus editissimus Montis Rocca; *M. Longa*, 6 August 1908, WU.—Monte Spinale; *FKL*, August 1863, WU.—Padon/Fassa; *M. de Sardagna*, 26 July 1882, WU.—Pfossental, Seitental des Schnalserthales, 7000–9000 Fuß; *Kerner*, s.d., WU.—Puflatsch; –, 8/82; ex Herb. Peyritsch IB-23982.—Rabbi-Joch (Tirol); –, Ende August 1900; Herb. E. Janchen W.—Rittneralpe; *Hausmann*, s.d., W.—Rittnerhorn; *Karl Grimus v. Grimburg*, July 1865[?], W.—Rittnerhorn (Unterhornhaus), 2044 m, grasige Triften (Porphyr). ; –, 30 July 1917, IB-15082.—S-Abhänge unter der Kor-Spitze b. Stilfser Joch, Tirol; *B. Kotula*, 2 August 1886, W.—Spitzhörndl bei Bruneck; *H. Schönach*, 22 August 1890, W.—steinige Triften am Sellajoch ober Plan; Grödnerthal.- Thonschiefer. 2200 m; *Preissmann*, 23 July 1904, W.—Stilfser Joch, 2600 m; *K. Wagner*, 25 August 1895, W.—Stilfserjoch; *Wettstein*, August 1893, WU.—Südtirol auf Urgestein auf dem Helm in Sexten; *Johann Vettl*, 26 July 1902, W.—Südtirol, Bindelweg am Pordoijoch; *Witasek bzw. Anton Klammer*, 1 August 1908, WU.—Südtirol, Vinschgau: SSE Tarsch, am Weg vom Tarscher Joch zur Pfaraalm (Jochpfarrer), Silikatfelsspalten, 2350 m.s.m.; *M. Pokorny + M. Strudl*, 26 July 1982, W.—Südtirol/Alto Adige, Dolomiten, Ghf. Seilbahn–Schönjöchl–Rifugio Plose–Pfannspitze (Monte Fana Grande), 2040–2542 m; *P. Schönswetter & A. Tribsch* (4907), 9 August 2000, WU.—Südtirol/Alto Adige, Ötztaler Alpen, Pfossental: Eishof (P. 2071)–Eisjöchl–Stettiner Hütte (Eisjöchlhütte)–S-Grat der Hochwilde, 2100–3000 m; *P. Schönswetter & A. Tribsch* (4922), 10 August 2000, WU.—Südtirol: Abzweigung Würzjoch-Mauraberghütte, ca. 2000–2100 m; lichter Nadelwald (Lärchen, Zirben), Silikat; *F. Krendl*, 30 July 1986, W.—Südtirol: Alpenmatten auf dem Puflatsch nächst Katelruth bei Bozen; *Johann Vetter*, 5 August 1927, W.—Südtirol: Cavalazza; *K. Ronniger*, 22 July 1908, W.—Suldenthal; *Michele de Sardagna*, 1877, WU.—Tirol Dreisprachenspitze am Stilfserjoch; *Witasek,* 21 August 1907, WU.—Tirol, Dolomiten, Nuvolau am Aufstieg von Caprile; *Ronniger*, 18 July 1904, W.—Trentino, Dolomiten, Passo Manghen–Monte Ziolera, 2050–2478 m; *P. Schönswetter & A. Tribsch* (3650), 27 August 1999, WU.—Trentino: Trento / Veneto: Belluno, Dolomiten, Pso. S. Pellegrino–Col Margherita, 1918–2545 m; *P. Schönswetter & A. Tribsch* (4643), 20 July 2000, WU.—Trentino: Trento, Dolomiten/Fleimstaler Alpen, Malga Cima d’Asta–Forcella Magna–Punta Socede–Rif. Ottone Brentari–Cima d’Asta, 2000–2847 m; *P. Schönswetter & A. Tribsch* (4661), 21 July 2000, WU.—Tyrolia centralis, Pustaria, Helm; *Schönach*, s.d., WU.—Veneto: Belluno / Friuli-Venezia-Giulia: Udine, Karnische Alpen, Rif. Calvi–Hochalpljoch–gegen den M. Peralba (Hochweißstein), 2200–2500 m; *P. Schönswetter & A. Tribsch* (4576), 4 July 2000, WU.—Veneto: Belluno, Karnische Alpen, Cra. di Coltrondo (ENE Kreuzbergpaß)–P. 2058 (Fort)–W-Grat des Col Quaternà, 1900–2400 m; *P. Schönswetter & A. Tribsch* (4589), 5 July 2000, WU.

#### Italy/Switzerland

Graubünden/Südtirol, Sesvenna-Gruppe, Poflwiesen W Reschenpaß–Seslat–Scharte SSW Piz Lad–Grubenjoch–Grüne Pleisen, 2100–2700 m; *Andreas Tribsch* (5068), 23 August 2000, WU.—Südtirol/Graubünden, Ortleralpen, Dreisprachenspitze (Cima Garibaldi) N Stilfser Joch–Rötlspitz (Punta Rosa), 2838–3026 m; *P. Schönswetter & A. Tribsch* (3677), 29 August 1999, WU.

#### Switzerland

Bernina, Cambrena Alta; *F. Morton*, 29 July 1923, W.—Bernina, Hte Engadin, Grisons; *Ros. Masson*, 1 August 1871, W.—Berninapaß: Alp Grün; *A. R. Paul*, July 1914, W.—Bündner Alpen; *M*… [?], s.d., W.—Canton Graubünden, Alpenmatten im obersten Sardascathal. (Silvrettagruppe); *K. Ronniger*, 7 August 1893, W.—Engadin, Piz Longlain; *Wettstein*, August 1899, WU.—Graubünden Samnaungruppe, Piz Arina; *Gerald Schneeweiss* (10643), 25 August 2004, WU.—Graubünden, Albula-Alpen, Champfèr–Alpe Suvretta–Scharte (P. 2870) SE Piz Güglia–Piz Güglia (Piz Julier), 2000–3381 m; *P. Schönswetter* (5083), 24 August 2000, WU.—Graubünden, Albula-Alpen, Fluela-Paß–Schwarzhorn, 2400–3147 m; *P. Schönswetter & A. Tribsch* (5064), 22 August 2000, WU.—Graubünden, Alpenweiden bei der Alpe Grüm im Puschlav; *Hayek*, 12 July 1908, WU.—Graubünden, bei Pontresina, auf Almtriften am oberen Schafbergszug, 2300 m; *A. Hirth*, 12 July 1927, W.—Graubünden, Schwarzhorn (Flüela), 2700 m; *K. Rechinger*, 28 July 1936, W.—Graubündten, Albulapass, Abhänge der Crestamara; *Ronniger*, 6 August 1900, W.—Helvetia. Albula; *Kerner com. Andees*, August 1866, WU.—Mons Bernina, Engadin; *Past*. *Rehsteiner*, August 1849, W.—Monte Camoghedo, Cima Ticino; *Alberto Franzoni*, 21 August 1857, W.—Mt. Bernina, Engadin super.; –, August; Herb. Pastor. Rehsteiner, Helvet W.—Nord/Tirol [wrong indication!], Steig von der Heidelbergerhütte zum Fimberjoch, 2450 m; *Paul Stark*, 8 August [19]34, IB-15094.—Ober-Engadin; ex Herb. Loritz[?], 1882, W.—Oberengadin beim Bernina-Hospiz, 2400; *Hayek*, 23 July 1908, WU.—Rhätische Alpen, Helvetia; *Regel*, s.d., W.—Val Bevers, Engadine Haute; *Muret*, Aout, W.—Verstanklateli; *M. Zoja*, 23 August 1918, WU.

### Senecio insubricus

#### Austria/Slovenia

in rupestribus montis Belscica in Alpibus Karavanken; *Paulin*, mense August, WU.—Kärnten/Gorenjska, Karawanken, Belscica, bei der Kote 1924, 1920 m; *Gerald M. Schneeweiß* (9679), 11 July 2003, WU.

#### Austria

auf dem Kamme des Sensen[?]joches in Tirol; *Fenzl*, 25 August 1861, W.—Aufstieg zum wilden Freyger; *Heinricher*, August 1923, IB-15089.—Felsen der Zischkelesspitze im Sellraintal; *Handel-Mazzetti*, 10 August 1896, WU.—Finstertalersee b. Kühtai; ex Herb. Peyritsch, s.d., IB-23981.—Mooserboden, Kaprun, Salzburg; *E. Vitek*, August 1974, W.—Moräne am Schwarzensteingl, 8000‘; *M.F. Müllner*, 27 August 1879, W.—Nord-Tirol, Brennergebiet, Trunerjoch Seite gegen Obernberg, steinig rasiger Hang, 2240 m; *Rudolf Berger*, 12 July 1921, IB-15095.—Nord-Tirol, Inzing: Roßkogl; Aufstieg von der Inzinger Alm zum See bei 1840 m; *Rudolf Berger*, 27 July 1923 , IB-15080.—Nord-Tirol, Nösslach; *Fritz Beer*, August 1905, IB-15100.—Nordtirol, Stubaythal in Tirol, am Gipfel des Schwarzhorns (2800 m); *Handel-Mazzetti*, 18 August 1898, WU.—Nord-Tirol: an Felsen am Südhang Rosskogel, Paiderspitze, Grieskogel, Hocheder im Sellraintal, auf Schiefer; *Handel-Mazzetti*, 14–15 September, WU.—Obernberger Joch bei Trins im Gschnitzthale, auf ca. 7000 Fuß; *Kerner*, 1870, WU.—Osttirol, Defereggen-Gebirge: von der Michelbachalm zur Weißen Wand; *F. Krendl*, 2 August 1969, W.—Ötztaler Alpen. Brunnenkogelhaus; *Erhard Knofel*, 3 September 1936, W.—Padasterjoch im Gschnitztal in Tirol; *Ruttker*[?], s.d., W.—Patscherkofel bei Innsbruck; *Kerner*, July 1875, WU.—Rotmoostal, unter dem Granatkogel, 2300 m, Obergurgl, Ötztal, Tirol, Österreich, Felsspalt; *E. Vitek*, 6 August 1975, W.—Stubaier Alpen: nahe der Dresdner Hütte, ca. 2400 m, Silikat; *H. Schönmann*, 14 July 1978, W.—Tirol, Gschnitztal, Leithenjoch; *Wettstein*, August 1895, WU.—Tirol, Ötztaler Alpen, Gaisbergtal (SSE Obergurgl): bis knapp vor den Gaisbergferner und östliche Talflanken (v. a. gegen P. 3049 südlich des Festkogels), 2200–3049 m; *P. Schönswetter & A. Tribsch* (3690), 30 August 1999, WU.—Tirol, Villgratner Berge, Kuschletalm–Pfanntörl–Toblacher Pfannhorn, 2000–2663 m; *P. Schönswetter & A. Tribsch* (4594), 5 July 2000, WU.—Tirol, Villgratner Berge, Lipper Alm–Kalksteiner Jöchl, 1900–2326 m; *P. Schönswetter & A. Tribsch* (4596), 5 July 2000, WU.—Tirol, Zillertaler Alpen, S unterhalb des Saurüssel, 2600–2700 m; *Peter Schönswetter & Katharina Bardy* (12129), 1 September 2007, WU.—Tirol. Am …grat bei der Dresdnerhütte, 2600 m; *Rud. Ronniger*, 11 August 1902, W.—Tirol. im kurzen Grase und im Gebüsch zwischen zwischen dem Schönbichele und dem bösen Weibele bei Lienz; *Johann Vetter*, 11 August 1922, W.—Tirol: Oberes Zillertal, Schlegeisgrund, Alluvionen des Schlegeisbaches zwischen Geröll, 1750 m; *Gerfried Leute*, 1–10 August 1976, W.—Tyrol, Schalderj.; *Tschurtschenthlr*., s.d., IB-15071.—von Lisens über das Horntaler Joch ins Stubai; –, s.d., IB-15087.—Kärnten, Karnische Alpen: E des Obstanser Sattels; 2500 m, E 12°29‘37‘‘ E 46°40‘39‘‘ N; *Peter Schönswetter* (14139), 10 May 2014, IB.

#### Austria/Italy

Pfitscher Joch; ex Herb. *Peyritsch*, 8 August 1881, IB-15070.—Pfitscher Joch; –, September 1883; Herb. Witting W.—Wolfendorn; ex Herb. Peyritsch, s.d., IB-44058.

#### Italy

Alpe Padon im Bellunesischen an der Gränze von Fassa; –, s.d.; Herb. Reichenbach fil. W.—Alpe Padon im Bellunesischen an der Gränze von Fassa; *Dr. Facchini*!, s.d., W.—Alpi della Pusteria; *L. Pacher,* s.d., WU.—auf den Lerbergl in Lappach, 2800 Meter; *Presser*[? Treffer?], 27 August 18[78?], WU.—auf Dolomitfelsen des Tierser Alpls bei Bozen; *J. Vetter*, 19 August 1906, W.—Belluno, Dolomiten, Passo Pordoi–Rifugio Sass Beccei–Col de Cuch–Sattel vor Cresta Larice, 2250–2563 m; *P. Schönswetter & A. Tribsch* (3642), 26 August 1999, WU.—Bergamasker Alpen, Alpi Orobie: Monte Legnone, am vom Rifugio Roccoli del Lorla zum Gipfel, 2200–2609, flachgründige Steinrasen, Silikat; exponierter Gratstandort; *E. Hörandl*, 27 July 1987, W.—Brescia, Gardaseeberge, Monte Colombine, 2200–2214 m; *P. Schönswetter & A. Tribsch* (3662), 28 August 1999, WU.—Dalle Valle Fassa; *Bernard*, July 1871, WU.—Fravort, Levico; *J. Schneider*, 24 July 1909, W.—in Neviss et Lappach, Tirol 2–2300 m; *G. Treffer*[?], 22 August 1880, W.—Jauffen, T; *J. Schneider*, 15 August 1910, W.—Lombardia: Como, Adula Alpen, (Bubegno–Il Sasso)–Croce di Campo–Cima Pianchette–Pizzo di Gino, 1739–2245 m; *Peter Schönswetter* (4984), 17 August 2000, WU.—Lombardia: Sondrio, Bernina-Alpen, (Pra Sücc)–Tre Cornini–Bivacco Bottani Cornaggio–P. 2636–gegen Monte Spluga, 2000–2700 m; *Andreas Tribsch* (4957), 14 August 2000, WU.—Lombardia: Sondrio/Lecco, Bergamasker Alpen, (Rifugio Roccoli dei Lorla)–Punta dei Merli–Bivacco Silvestri–Sentiero d’Angeli–Monte Legnone–S-Grat des Monte Legnone, 2000–2609 m; *P. Schönswetter & A. Tribsch* (4969), 15 August 2000, WU.—Monte Colombine, in den Brescianer Alpen, 1800–2000 m; *Mandl*, July 1960, W.—Oberitalien: auf einem Fels, am Aufstieg zum Lago d’Ritorto; *K. Ronniger*, 2 August 1908, W.—Padon/Fassa; *M. de Sardagna*, 26 July 1882, WU.—Pfitscher Joch bei Sterzing, Schiefer, ca. 2200 m; *Handel-Mazzetti*, 31 July 1899, WU.—Pfossental im Vinstgau, am Pfasereck; *Handel-Mazzetti*, 26 August 1907, WU.—Rabbi-Joch (Tirol); –, Ende August1900; Herb. E. Janchen W.—Rittnerhorn (Unterhornhaus), Tirol, 2044 m, grasige Triften (Porphyr); –, 30 July 1917; ex Herb. Diettrich-Kalkhoff IB-15101.—Rittnerhorn, 2200 m; *F. Petz*, July 1916, IB-15078.—Schlern, unter den Rosszähnen, Caminthal; *Heinricher*, 12 July 1901, IB-15081.—Schnalser Tal; *Karl Grimus v. Grimburg*, August 1861, W.—Südtirol, Jaufen-Pass/Passo Giove. Alto-Adige; *R. Seipka*, 2 September 1973, W.—Südtirol, Jauffenpass Südseite, 2105 m; *E. Vitek*, 1 September 1975, W.—Südtirol, Rittnerhorn, Unterhornhaus, 2044 m; *Diettrich-Kalkhoff*, 30 July 1917, IB-15101A.—Südtirol. An Felsen auf dem Rabbijoch; *Joh. Vetter*, 23 August 1906, W.—Südtirol. Dolomiten auf den Bergen bei Deutschnofen; *Dr. Urban,* 11 August 1913, W.—Südtirol/Alto Adige, Dolomiten, Ghf. Seilbahn–Schönjöchl–Rifugio Plose–Pfannspitze (Monte Fana Grande), 2040–2542 m; *P. Schönswetter & A. Tribsch* (4907), 9 August 2000, WU.—Südtirol/Alto Adige, Ötztaler Alpen, Pfossental: Eishof (P. 2071)–Eisjöchl–Stettiner Hütte (Eisjöchlhütte)–S-Grat der Hochwilde, 2100–3000 m; *P. Schönswetter & A. Tribsch* (4922), 10 August 2000, WU.—Südtirol/Alto Adige, Sarntaler Alpen, Schalderer Tal: Aufstieg von E zur Schalderer Scharte–Schalderer Scharte–Schrotthorn (Corno del Ceppo), 2000–2590 m; P. *Schönswetter & A. Tribsch* (4919), 9 August 2000, WU.—Südtirol/Alto Adige, Zillertaler Alpen, Obere Neveser Alm–Neveser Joch–Tristentaler See–(weglos auf die) Tristenspitze, 2150–2716 m; *P. Schönswetter & A. Tribsch* (4898), 8 August 2000, WU.—Südtirol: Dolomiten, Plose, Gipfelregion; *K. Ronniger*, 25 July 1912, W.—Südtirol: Zillertaler Alpen: W Sand im Taufer, von der Bergstation Speikboden zum Speikboden, ca. 1960–2500 m, Almmatten, Felsheiden, Felsen, Silikat; *F. Krendl*, 1 August 1986, W.—Tirol, Rabbijoch bei Meran; *Fritz Beer*, August 1911, IB-15099.—Tirol, Seebergl, Lappach; *G. Treffer*, 27 August 1878, WU.—Tirol. Steiniger Boden am Rabbijoch (2500 m); *Dr. Korb*, 22 August 1906, W.—Tirol. Stilfserjoch; *Kamerlander*, 1872, WU.—Tirol-auf Faßer Alpen; *Gandalf*, 6 August 180[?], WU.—Trentino, Dolomiten, Passo Manghen–Monte Ziolera, 2050–2478 m; *P. Schönswetter & A. Tribsch* (3650), 27 August 1999, WU.—Trentino: Trento, Dolomiten/Fleimstaler Alpen, Malga Cima d’Asta–Forcella Magna–Punta Socede–Rif. Ottone Brentari–Cima d’Asta, 2000–2847 m; *P. Schönswetter & A. Tribsch* (4661), 21 July 2000, WU.—Trentino: Trento, Dolomiten/Fleimstaler Alpen, Pso. Rolle–Tognazza–Cavallazza Piccola–W-Flanke der Cavallazza Piccola, 2000–2310 m; *P. Schönswetter & A. Tribsch* (4650), 21 July 2000, WU.—Trentino-Alto Adige: Trento, Adamello-Presanella, Val Folgorida (Val Genova W Pinzolo)–Malga Folgorida–gegen den Passo delle Toppette, 2300–2750 m; *P. Schönswetter & A. Tribsch* (5032), 20 August 2000, WU.—Karnische Alpen: Hochspitz, am Karnischen Höhenweg SSW des Gipfels; 2375 m, E 12°39‘57‘‘ N 46°39‘16‘‘; *Peter Schönswetter* (14138), 10 May 2014, WU.

#### Switzerland

Ticino, Lepontische Alpen (Tessiner Alpen), Mornera (NW Bellinzona)–Capanna Albagno–Cima dell’Uomo, 1800–2390 m; *P. Schönswetter & A. Tribsch* (5114), 28 August 2000, WU.

### Senecio noricus

#### Austria

[Steiermark], Preberrücken bei Krakauebene, zwischen Steinen und im Gerölle, Urgebirge, 2600 m; *J. Genta*, 23 July 1931, W.—Alpe Kühlbrein bei Turrach, Steiermark; *Jos. Ahrenberger*, July 1867, W.—Alpen Niedere Tauern, Judenburger Alpen, Zinggen; *Fenzl*, s.d., W.—auf dem Plateau des Speiereck; *Louis Keller*, 29 August 1895, W.—auf dem Preber; *M. F. Müllner*, s.d., W.—bei Mallnitz: auf Alpentriften am Aufstieg vom Seebachthal Hannoverhaus und in dessen Umgebung, 2300 m; *Hirth*, 12 August 1924, W.—Großglockner in Kärnthen; *Rainer Graf*, s.d., W.—Hohe Tauern, Goldberg Gruppe: In monte Schareck prope Böckstein, in ca. 2900–3100 m; *Patzak*, September 1954, W.—Hohe Tauern. Hochalmspitzgruppe. Säuleck. Knapp unter dem Gipfel; *Schönbeck*, 6 August 1948, WU.—in vicinis des Kalser Törls; *Pappitz*, 12 August 1820, W.—Kärnten, Hohe Tauern: Ankogelgruppe, Hannover Hütte–Gipfel des Ankogel, 2600–3246 m; *P. Schönswetter & A. Tribsch* (3770), 14 August 1999, WU.—Kärnten, Hohe Tauern: Goldberggruppe, Sadniggruppe, Grossfragant, Sadnigscharte, 2480 m; *Gerald M. Schneeweiß* (11148), 6 July 2006, WU.—Kärnten, Hohe Tauern: Hafnergruppe, Krumpenkar (ENE der Wastlbauerhütte)–Marschneid–Hafner, 2000–3076 m; *P. Schönswetter & A. Tribsch* (3759), 21 August 1999, WU.—Kärnten, Hohe Tauern: Kreuzeckgruppe, (Ghf. Bergheimat -) Ochsnerhütte–Scharnik (über SW-Grat)–Gursgentörl–Lamnitzsee–Torwand, 1850–2657 m; *P. Schönswetter & A. Tribsch* (4613), 7 July 2000, WU.—Kärnten, Spitze des Hocheck im Mölltal; *J. Witasek*, 12 August 1899, WU.—Kärnthner Alpen; –, July August, WU.—Kl. Fleiss, Heiligenblut; *J. Schneider*, 26 August 1928, W.—Knappenloch am Goldberg; ex Herb. Peyritsch, s.d., IB-23983.—Lungau (Salzburg), Bundschuh; *F. Vierhapper,* s.d., WU.—Lungau (Salzburg), Weißbriach; *F. Vierhapper*, ca. 1900, WU.—Millstädter Alpe Gipfelbereich; *M. A. Fischer*, 22 July 1966, WU.—Moschelitzen; *M. A. Fischer*, 25 July 1966, WU.—Nassfelder Tauern; *Ruprecht*, s.d., WU.—N-Kärnten: Gurktaler Alpen, Kleiner Speckkofel bei St. Lorenzen, 2097 m; *K. Ronniger*, 26 July 1909, W.—N-Kärnten: Hochalmgruppe, Großelend oberhalb der Osnabrückerhütte; *K. Ronniger*, 30 July 1907, W.—Pinzgau, am Kapruner Thörl; *Karl Aust*, Sommer 1883, W.—Schober im Lessachwinkel im Lungau; *F. Vierhapper*, 4 August 1959, WU.—Schoberspitze im Eselsberggraben bei Pöllau, Urgebirge, 2200 m; *Schamperl*, 15 July 1931, W.—Schwarzkopf in Fusch, Pinzgau; *Carl von Sonklar*, August 1862, WU.—Steiermark, Niedere Tauern, Seckauer Zincken, Aufstieg vom Pabstriegel über Kote 1869 zum Gipfel, 1950–2330; *F. Kummert*, July 1971, W.—Steiermark, Wölzer Tauern, Grillerlucke, 2150 m; *Gerald M. Schneeweiß & Peter Schönswetter* (12467), 21 August 2008, WU.—Steiermark, Zinken bei Seckau: 1900 m, Granit; *Leo Derganc*, 19 August 1904, W.—Stiria superior. In graminosis montis Großer Bösenstein prope Hohentauern; 2250–2150 mt. s.m., solo schistaceo; *Pernhoffer*, s.d., WU.—Tirol, Schleinitz bei Lienz, auf den höchsten Jochen in Felsen und Spalten; *Th. Pichler*, 5 September, W.—Trübeck-Feldeck, 2350–2500, Niedere Tauern; *F. Buxbaum*, 27 June 1920, W.

### Senecio disjunctus

#### Austria

[Steiermark], Preberrücken bei Krakauebene, zwischen Steinen und im Gerölle, Urgebirge, 2600 m; *J. Genta*, 23 July 1931, W.—Alpe Kühlbrein bei Turrach, Steiermark; *Jos. Ahrenberger*, July 1867, W.—am Hohen Zinken bei Seckau; *H.W. Reichardt*, 12 August 1861, W.—auf dem Gipfel des Hochgolling, 9050‘; *O. Simony*, 9 August 1863, W.—auf dem Plateau des Speiereck; *Louis Keller*, 29 August 1895, W.—auf Fels und im Gerölle der Judenburger Alpen; *Gebhard, Welwitsch*, August, W.—Aufstieg zur Rinsennnock und über den Grat zur Kornock, Almwiese auf dem Grat, zwischen Felsbrocken, 2330 m; *B. Zimmer*, 5 August 2006, W.—bei Schladming: Gollinghütte, obere Steinwänderalm; *K. Ronninger,* 30 July 1916, W.—Eisenhut (Häufig) 1904, Hochreichert (Gipfel 2417m) nied. Tauern, 1905; *Dr. Hel. Zellner*, s.d., W.—Gipfel des Steinkarzink bei Schladming, Steiermark; *Zahlbruckner, Seidlesberger*, 30–31 July 1887, W.—Gipfelblock des Gr. Bösenstein, 2449, Gneis; *Heinrich*, 14 August 1911, W.—Grosser Bösenstein bei Dorfe „Hohentauern“ in den Rottenmanner Tauern, 2400 m; *Pernhofer*, 28 August 1880, W.—Großer Bösenstein bei Hohentauern; *Pernhofer*, 20 August 1880, WU.—Großer Bösenstein beim Dorfe „Hohentauern“; *Pernhofer*, 28 August 1880, WU.—Hohenwart, Niedere Tauern, Steiermark; *Julius Nevole*, August 1906, WU.—Judenburgeralpen und Kärnthner Alp.; *Gebh. Moser*[?], s.d., W.—Kleinalpe, 6200‘; *Fürstenwerther*, 30 July 1854, W.—Knallstein in der Sölk, Glimmerschiefer; *J. B*, 10 August 1868, WU.—Lungau (Salzburg), Hochgolling; *F. Vierhapper*, ca. 1900, WU.—Niedere Tauern, Bösenstein, knapp unter Gipfel auf Wiese, Aufstieg vom Scheiblingsee zum Gipfel/Ostalpen, Niedere Tauern, Aufstieg zum Bösenstein von der Edelrautehütte, Almboden; *E. Schönbeck/E. Temesy*, 29 July 1956/10 August 1952, WU.—Niedere Tauern. Hochgolling; *Erwin Janchen*, 3 August 1912, WU.—Paturages du terrain argileux sur le micaschiste de la montagne Rottenmanner Tauern, a 2000 metres, dans les alpes de la Haute Styrie; *Rec. Oberleitner*, 21 July 1868, W.—Pâturages du terrain argileux sur le micaschiste de la montagne Rottenmanner Tauern, à 2000 mètres, dans les alpes de la Haute Styrie; *Oberleitner*, s.d., IB-15075.—Preber, Tamsweg, 2400 m, Salzburg; *J. Schneider*, 22 August 1902, W.—Rottenmanner Tauern: Hochhaide; *E. Hübl*, s.d., W.—Salzburg, im Schiefergerölle auf den Abhängen des Hochgolling; *Johann Vetter*, 23 July 1915, W.—Salzburg, Lungau, Niedere Tauern, Landwirseenkessel, zwischen ob. Zurgriegel und L[andawirsee]Hütte, 1900 m, Urgestein; *G. Cufodontis*, 15 July 1929, W.—Salzburg, Niedere Tauern: im Gestein unterm Gipfel der Seekarspitze am Radstätter Tauern; *Ernst Korb*, 16 August 1932, W.—Schladming Grat Rettingscharte, Kieseck; *R. Eberwein*, 5 August 1902, WU.—Schladminger Tauern, Sonntagskaar, beschränkt auf eine kleine Stelle; *O. Widmann*, 20 July 1924, W.—Seckauer Zinken, n Steyermark, Gneiss; *Wolosrzczak*, 4 August 1876, W.—Seckaueralpen; *Haß*..ann, s.d., W.—Steiermark Zinken bei 2000 m Granit; *Leod. Derganc*, August 1895, WU.—Steiermark, Gurktaler Alpen, Dieslingsee–(Großer und Kleiner) Eisenhut, 1850–2441 m; *P. Schönswetter & A. Tribsch* (4557), 22 June 2000, WU.—Steiermark, Niedere Tauern, Seckauer Zincken, Aufstieg vom Pabstriegel über Kote 1869 zum Gipfel, 1950–2330; *F. Kummert*, July 1971, W.—Steiermark, Planneralpe: vom Plannereck zum Hochrettensten; *F. Krendl*, 13 July 1968, W.—Steiermark, Rottenmanner Tauern, Bösenstein, Alpenmatten, 1200–1800 m; *K.H. Rechinger*, August 1953, W.—Steiermark, Schladminger Tauern, markierter Weg von der Wödlhütte zur Hochwildstelle: E-Flanke des Gruberberg–Neualmscharte–Hochwildstelle, 1900–2747 m; *P. Schönswetter & A. Tribsch* (3804), 5 September 1999, WU.—Steiermark, Schladminger Tauern, Umgebung der Samspitze (NNE der Landwirseehütte), 2320–2381 m; *P. Schönswetter & A. Tribsch* (3798), 4 August 1999, WU.—Steiermark, Wölzer Tauern, Schießeck: N-Flanke des Gipfels (mit Kreuz), 2260 m; *Gerald M. Schneeweiß & Peter Schönswetter* (12465), 21 August 2008, WU.—Steiermark, Zinken bei Seckau (Granit); *Leod. Derganc*, August 1894, WU.—Steiermark, Zinken bei Seckau: 1900 m, Granit; *Leo Derganc*, 19 August 1904, W.—Steiermark. Auf dem Hochzinken bei Seckau; *Thomas Pichler*, *comm. Dr. v. Eichenfeld*, s.d., W.—Steinige Matten der Berge im Liegnitz; *F. Vierhapper*, August 1899, WU.—Stiria, Rottenmanner Tauern; *Gebhard*, s.d., W.—Stiria superior. In graminosis et petrosis montis Zinken prope Sekkau; 2200–2390 mt. s.m., solo schistaceo; *Pernhoffer*, s.d., WU.—Stiria, Hoher Zinken Altitudo 5000’. Im Gerölle des Glimmerschiefers; *Eq. de Pittoni*, s.d., W.—Stiria: in Monte Zinken; *H. Beck*, August 1880, W.—von der Steinwänder Alp (Schladmingthal) bis auf den Gipfel des Hochgolling, 6000–9050’; *O. Simony*, 9 August 1863, W.—Zinken; *Fenzl*, s.d., W.

#### Italy

Corno Nero, Cavalese; *M. de Sardagna*, 1862, WU.—Dreisprachenspitze; –, s.d.; ex Herb. Peyritsch IB-44056.—felsige Abhänge an der Dreisprachenspitze des Stilfserjoches. Glimmerschiefer, 2800 m; *E. Preissmann*, 23 July 1901, W.—Habitat pascua alpina JochGrim[m]; *Sarnthein*, anno 1807 mense August, WU.—im Madritschtal von 7500 bis gegen 8500’; *O. Simony*, 1855, W.—Lombardia: Brescia, Gardaseeberge, Rifugio C. Tassara (NW Pso. Croce Domini)–Malga Val Fredda–Passo di Val Fredda–Terre Fredde NE Monte Frerone, 2000–2400 m; *P. Schönswetter & A. Tribsch* (5025), 19 August 2000, WU.—Lombardia: Sondrio/Brescia, Sobretta-Gavia-Gruppe, Passo Gavia–gegen den Monte Gavia, 2630–2900 m; *Andreas Tribsch* (5017), 18 August 2000, WU.—Monte Tonale; *M. de Sardagna*, 19 August 1879, WU.—Mt. Scorluzzo bei 9000’; –, 8 August 1885; ex Herb. Peyritsch IB-15088.—Sondrio/Brescia, Ortleralpen, Passo di Gavia: entlang der Straße von 0,6 km S der Paßhöhe bis 1 km NE der Paßhöhe, 2500–2618 m; *P. Schönswetter & A. Tribsch* (3664), 28 August 1999, WU.—Stilfser Joch–Dreisprachenspitze, Triften, 2800 m; –, *August* 1907. Ex herb. Diettrich-Kalkhoff, IB-15083.—Südtirol. Rosetta bei S. Martino di Castrozza; *F. Lemperg*, 16 July 1935, W.—Tirol, Rabbijoch bei Meran; *Fritz Beer*, August 1911, IB-15096.—Trentino: Presanella: vom Lago Lambini zum Lago Ritorto, 2055–2300 m; Felstriften, Tonalit; *W.Burri & F. Krendl*, 20 September 1983, W.—Trentino: Trento, Dolomiten/Fleimstaler Alpen, Malga Cima d’Asta–Forcella Magna–Punta Socede–Rif. Ottone Brentari–Cima d’Asta, 2000–2847 m; *P. Schönswetter & A. Tribsch* (4664), 21 July 2000, WU.—Trentino-Alto Adige: Trento, Adamello-Presanella, Val Folgorida (Val Genova W Pinzolo)–Malga Folgorida–gegen den Passo delle Toppette, 2300–2750 m; *P. Schönswetter & A. Tribsch* (5032), 20 August 2000, WU.—Umbrail; –, s.d.; ex Herb. Peyritsch IB-44057).

#### Italy/Switzerland

Südtirol/Graubünden, Ortleralpen, Dreisprachenspitze (Cima Garibaldi) N Stilfser Joch–Rötlspitz (Punta Rosa), 2838–3026 m; *P. Schönswetter & A. Tribsch* (3677), 29 August 1999, WU.
